# Non-Replicating *Mycobacterium tuberculosis* Elicits a Reduced Infectivity Profile with Corresponding Modifications to the Cell Wall and Extracellular Matrix

**DOI:** 10.1371/journal.pone.0087329

**Published:** 2014-02-06

**Authors:** Joanna Bacon, Luke J. Alderwick, Jon A. Allnutt, Evelina Gabasova, Robert Watson, Kim A. Hatch, Simon O. Clark, Rose E. Jeeves, Alice Marriott, Emma Rayner, Howard Tolley, Geoff Pearson, Graham Hall, Gurdyal S. Besra, Lorenz Wernisch, Ann Williams, Philip D. Marsh

**Affiliations:** 1 Public Health England, Microbiology Services, Porton Down, Salisbury, Wiltshire, United Kingdom; 2 Institute of Microbiology and Infection, School of Biosciences, University of Birmingham, Birmingham, United Kingdom; 3 MRC Biostatistics Unit, Institute of Public Health, Cambridge, United Kingdom; University of Delhi, India

## Abstract

A key feature of *Mycobacterium tuberculosis* is its ability to become dormant in the host. Little is known of the mechanisms by which these bacilli are able to persist in this state. Therefore, the focus of this study was to emulate environmental conditions encountered by *M. tuberculosis* in the granuloma, and determine the effect of such conditions on the physiology and infectivity of the organism. Non-replicating persistent (NRP) *M. tuberculosis* was established by the gradual depletion of nutrients in an oxygen-replete and controlled environment. In contrast to rapidly dividing bacilli, NRP bacteria exhibited a distinct phenotype by accumulating an extracellular matrix rich in free mycolate and lipoglycans, with increased arabinosylation. Microarray studies demonstrated a substantial down-regulation of genes involved in energy metabolism in NRP bacteria. Despite this reduction in metabolic activity, cells were still able to infect guinea pigs, but with a delay in the development of disease when compared to exponential phase bacilli. Using these approaches to investigate the interplay between the changing environment of the host and altered physiology of NRP bacteria, this study sheds new light on the conditions that are pertinent to *M. tuberculosis* dormancy and how this organism could be establishing latent disease.

## Introduction

Tuberculosis (TB) is characterised by long term persistence in a latent state, which after decades, can be reactivated and lead to further spread of the disease. *Mycobacterium tuberculosis* is thought to adapt and thrive in diverse environmental niches *in vivo* during latency [1]. However, the location and physiology of the bacterium during this phase of the disease remains unclear [2] [3]. It is generally believed that during latency *M. tuberculosis* resides within the solid granulomas which are characteristic of latent TB infection. It is thought that the tubercle bacilli located in these regions reside in a slow growing or non-replicating dormant-like state, which could be achieved by exposure to perturbations in the availability and supply of oxygen and the sources of available nutrients [1] [4]. The dormant-like state has been extensively investigated using *in vitro* models in an attempt to simulate the granuloma environment with a particular focus on hypoxia-induced non-replicating persistent (NRP) states, which have demonstrated that *M. tuberculosis* is able to survive for extended periods of time [5] [6]. The DosR regulon is implicated in the hypoxic adaptation of *M. tuberculosis* and subsequent virulence profiles. During infection studies, *M. tuberculosis dosR* mutants exhibit a variably attenuated phenotype [7] [8] [9]. Differences in the methodologies employed to study *M. tuberculosis* dormancy such as the choice of animal species, the disease-stage, and the parameters used to define attenuation, make interpretation of these variable findings difficult to reconcile. However, these studies highlighted the fact that there are other environmental factors, such as the availability of nutrients, which could be triggers for establishing latent TB infection [1].

The focus of this study was to model environmental conditions other than hypoxia, such as nutrient-depletion, that will be encountered by *M. tuberculosis* during chronic infection. We exploited the advantages of controlled batch fermenter cultures of *M. tuberculosis* utilising fatty acids as the primary carbon source, which were gradually depleted over an extended period of time. The physiological and pathogenic responses of *M. tuberculosis* to nutrient depletion have not been investigated fully in previous studies. Therefore, the role of cell wall re-modeling in the establishment of NRP and the impact of these different physiological states on infectivity in the guinea pig were explored.

## Results

### NRP derived by nutrient depletion

**Figure 1:**
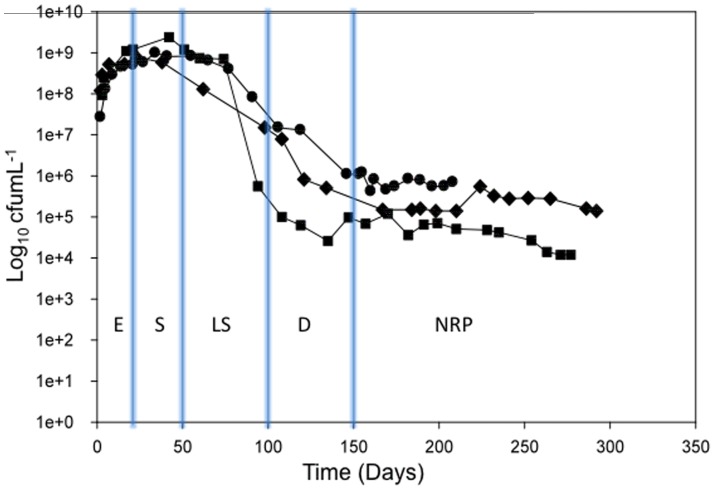
Growth curves for *M. tuberculosis* grown in medium containing Tween 80 as the primary carbon source. Total viable counts for Culture 1 (circles), Culture 2 (diamonds), and Culture 3 (squares), were measured over an extended period of at least 200 days. An estimation of each growth phase is indicated on the graph: exponential phase (E), stationary phase (S), late stationary phase (LS), death phase (D), and non-replicating persistent phase (NRP).

Three independent replicate cultures of *M. tuberculosis*, (Cultures 1, 2, and 3) were established using a fermenter-controlled batch growth model based on a modified continuous culture vessel that we have previously reported [10]. This approach has enabled us to establish non-replicating persistent (NRP) populations of *M. tuberculosis* in culture by the gradual depletion of nutrients over time in a controlled oxygen-replete environment. The medium was CMM Mod6 (medium recipe, Table S1, supporting information), which contains the primary carbon source Tween 80; hydrolysis to oleate provided an indirect source of fatty acids [11] [12]. The viability of organisms was monitored over an extended period of at least 200 days. In each of the three independent experiments, exponential phase (E) occurred during the first 10 days of culture (total viable counts reached approximately 10^9^ cfu mL^−1^) and proceeded into stationary phase (S) which lasted for approximately 40 days (Figure 1). Over the subsequent period, cells remained in a late stationary phase (LS) followed by a significant drop of 10^3^ cfu mL^−1^ in cell count viability (D) (Figure 1). A final phase, that we termed NRP, lasted for a period of at least 60 days with a concomitant viable count of 10^5^–10^6^ cfu mL^−1^ (Figure 1). Multiple culture samples were removed at growth stages E, S, LS, and NRP and used for subsequent physiological and biochemical analyses. The death phase (D) was not included in the analyses. There was a finite amount of biomass from each time-course and so each type of analysis could not be performed on all three cultures. For clarification, Culture 1 was sampled for transcriptomics, Culture 2 was sampled for transcriptomics and lipid/carbohydrate analyses, and Culture 3 was used for lipid/carbohydrate analyses and infectivity studies. Tween 80 was confirmed as depleted and restricting growth by the analysis of free fatty acids in the spent culture supernatant; eighty percent of the Tween 80 was consumed by the NRP phase for both Culture 2 and Culture 3 (Figure S1, supporting information). Tween 80-depletion was limiting growth as shown by the addition of Tween 80 (0.2% v/v in water) to a further NRP culture, which initiated re-growth over a period of 12 days and viability levels rose to 10^8^ cfu ml^−1^ (unpublished results).

### The morphology of M. tuberculosis isolated from each growth phase

**Figure 2:**
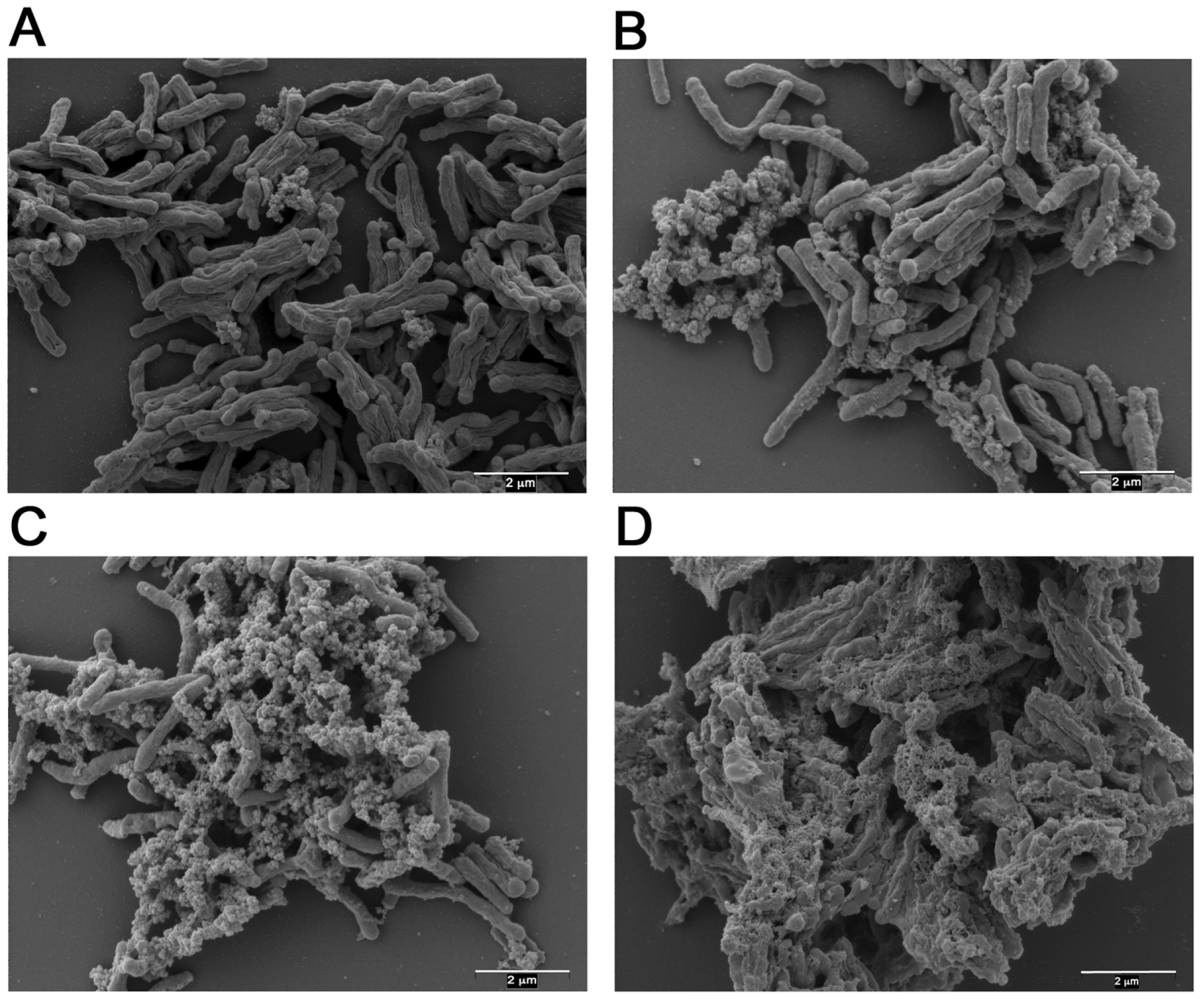
Scanning electron micrograph images of formaldehyde-fixed cells sampled from Culture 2. Panel A) exponential phase at day 8; Panel B) stationary phase at day 44; Panel C) late stationary phase at day 107; and Panel D) NRP at day 292.

**Figure 3:**
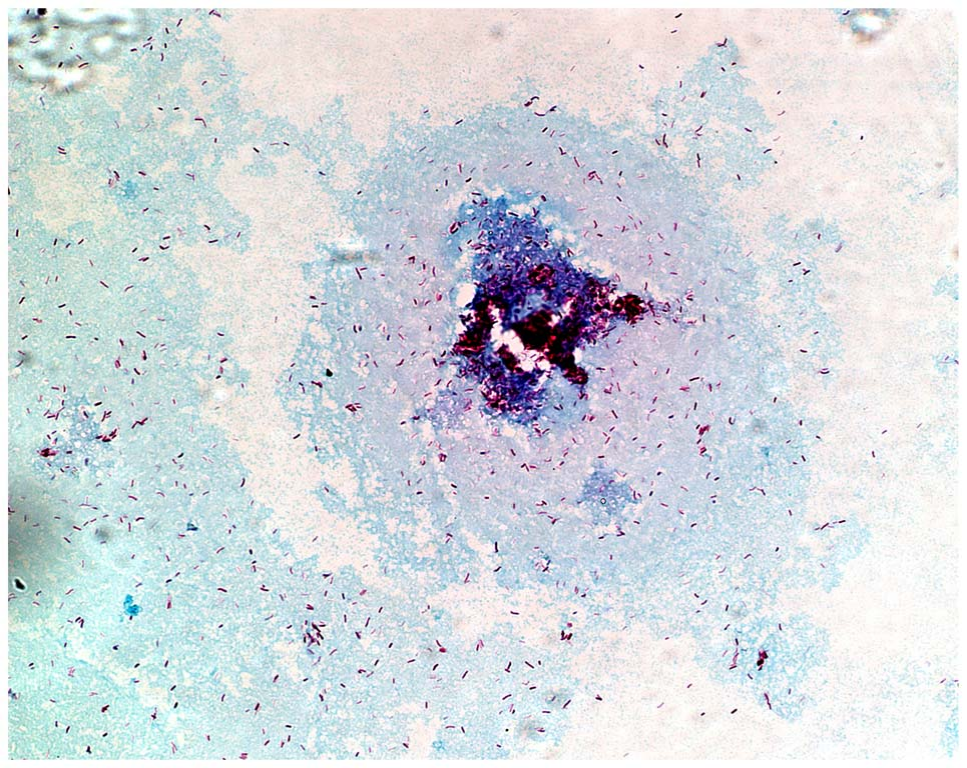
Cells sampled during late stationary phase. Bacilli stained with Ziehl Neelson and the surrounding extracellular material was stained with Alcian Blue, pH×100.

Electron microscopy revealed the presence of extracellular material surrounding *M. tuberculosis* cells sampled from each of the growth phases (Figure 2), which was particularly pronounced in late stationary phase and NRP phase (Figure 2C and D). Samples from late stationary phase were stained with either Alcian Blue or Sudan Black in order to selectively identify the presence of carbohydrates or lipids, respectively [13]. In each case, the Ziehl-Neelsen acid fast stain was used in parallel to detect *M. tuberculosis* bacilli. Staining with Sudan Black gave a negative result for each of the samples tested, which suggested that the extracellular material was unlikely to consist predominantly of lipid. However, upon staining with Alcian Blue followed by visualisation using light microscopy, at x100 magnification, we observed a clear selective blue-coloration of the extracellular material surrounding the *M. tuberculosis* bacilli (Figure 3). These initial findings indicated that, during the latter phases of growth, these cultures over-produced an extracellular material which is, at least in part, composed of carbohydrate, which is a characteristic of biofilm formation in bacteria survival. The content of the following sections were focused on the analyses of the extracellular material and the re-modeling of the cell wall as these have implications for the survival of *M. tuberculosis* in NRP.

### Re-modeling of the cell wall

**Figure 4:**
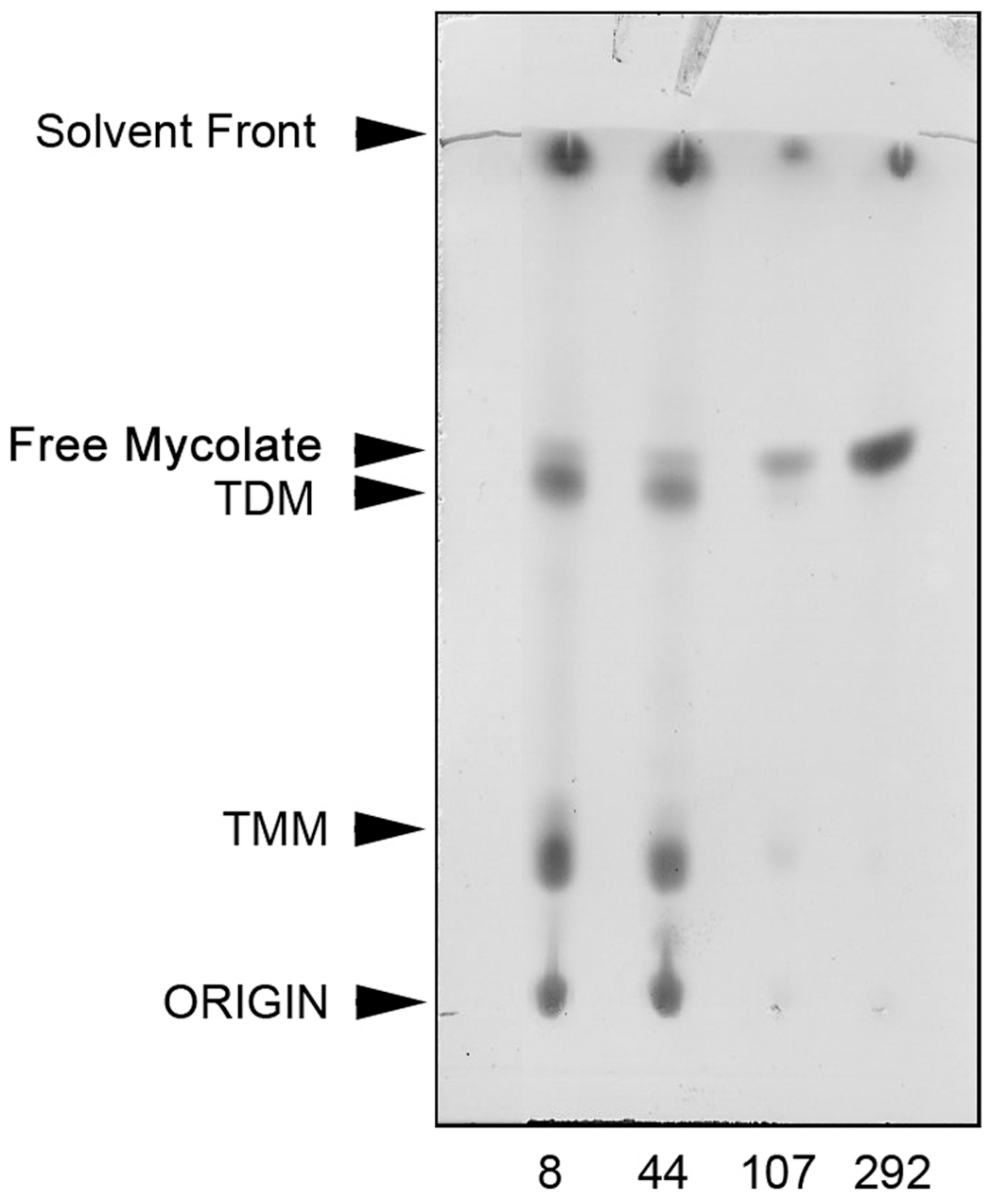
1D TLC of freely extractable lipids from Culture 2. Cell biomass was sampled during exponential phase (day 8), stationary phase (day 44), late stationary phase (day 107) and non-replicating persistent phase (day 292). Lipids migrating on the TLC were visualised by staining with MPA and compared to known standards [14].

**Figure 5:**
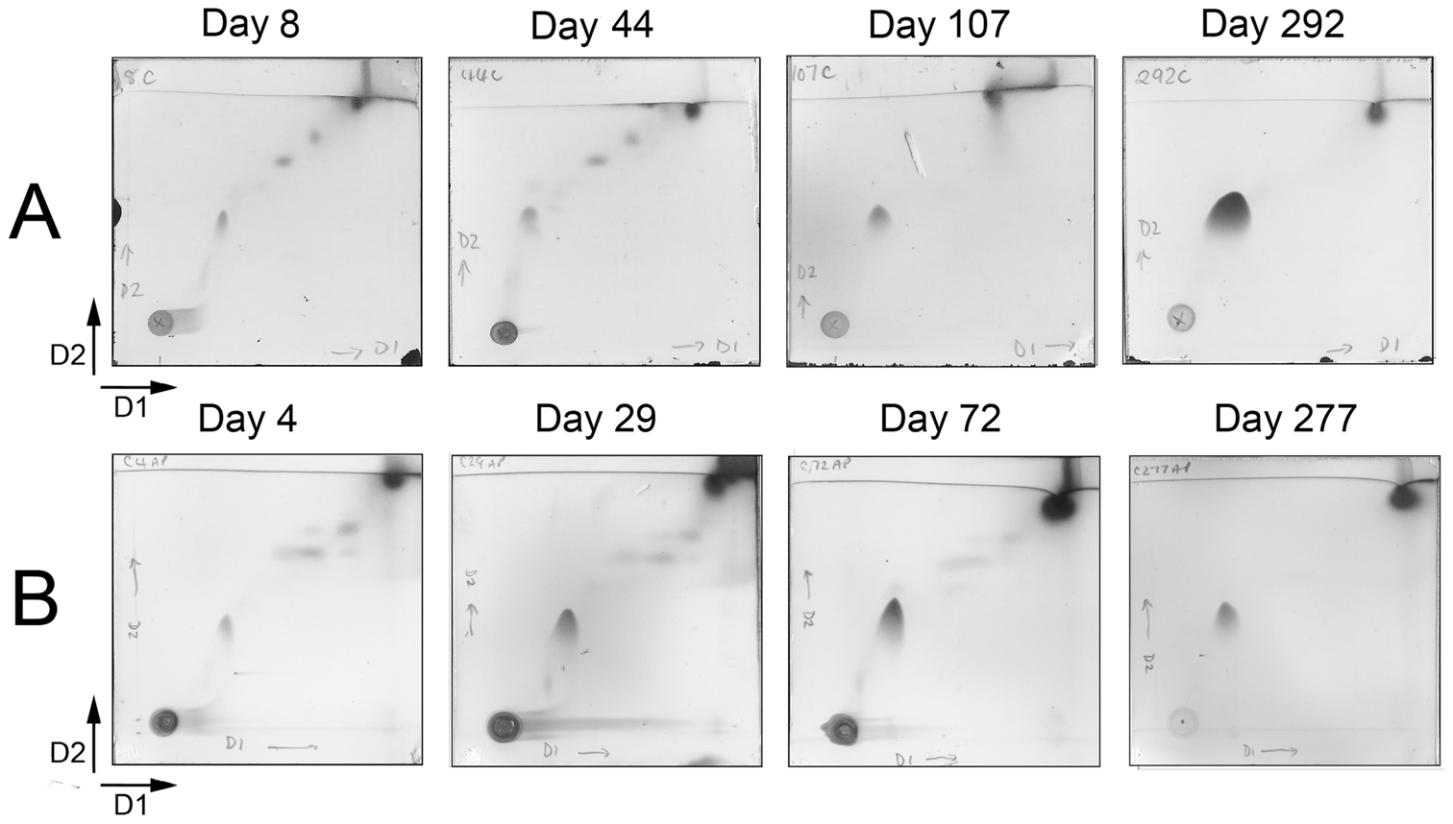
2D TLC analyses of apolar lipids extracted from Culture 2 and Culture 3. Panel A, cell biomass was sampled from culture 2 during exponential phase (day 8), stationary phase (day 44), late stationary phase (day 107), non-replicating persistent phase (day 292). Panel B, cell biomass was sampled from culture 3 during exponential phase (day 4), stationary phase (day 29), late stationary phase (day 72), NRP phase (day 277). Lipids migrating on the TLC (using solvent system C) were visualised by staining with MPA and compared to known standards [14].

Mycobacteria have an unusual lipid-rich cell wall, which is complex in structure and vital for cell survival and host-pathogen interactions. Polar and apolar lipids were selectively extracted using organic solvents from cells that had been harvested from each of the four phases of growth, E, S, LS, and NRP (Figure 1). Lipid fractions were analysed using thin layer chromatography (TLC) to detect individual species of cell wall lipids as compared to known standards [14]. 1D TLC analyses of freely extractable lipids from Culture 2 are illustrated (Figure 4) and show a gradual increase in the cellular content of free mycolate and a corresponding depletion in the content of trehalose dimycolate (TDM) and trehalose monomycolate (TMM). The data are representative of Cultures 2 and 3. Apolar lipids extracted from Culture 2 and Culture 3 were subjected to 2D TLC analysis to enable further comparisons of how the lipid profile alters at each growth stage over the duration of the culture (Figure 5). There was a significant accumulation of a spot on each TLC plate throughout the time-course (using solvent system C) which migrated to a position corresponding to free mycolic acid (MA) [14], for Culture 2 and Culture 3 (Figure 5). In order to quantify this change in lipid content over time, we conducted a densitometry analysis of the fast-migrating spots on the TLC (corresponding to MA) normalised against the non-migrating lipids at the origin of each TLC plate. For each TLC (representing the day at which samples were collected from Culture 2), the ratio of lipids (origin: mycolate) increased gradually as follows, day 8 (1∶0.95), day 44 (1∶0.96), day 107 (1∶0.99) and day 292 (1∶1.27). We also observed an almost identical pattern of MA accumulation in Culture 3, as demonstrated by the following increase in the ratio of lipids (origin: mycolate), day 4 (1∶0.75), day 29 (1∶0.90), day 72 (1∶0.92) and day 277 (1∶1.05). No changes in any of the other lipids were found [14].

### Adaptation through alterations in cell wall carbohydrates

**Figure 6:**
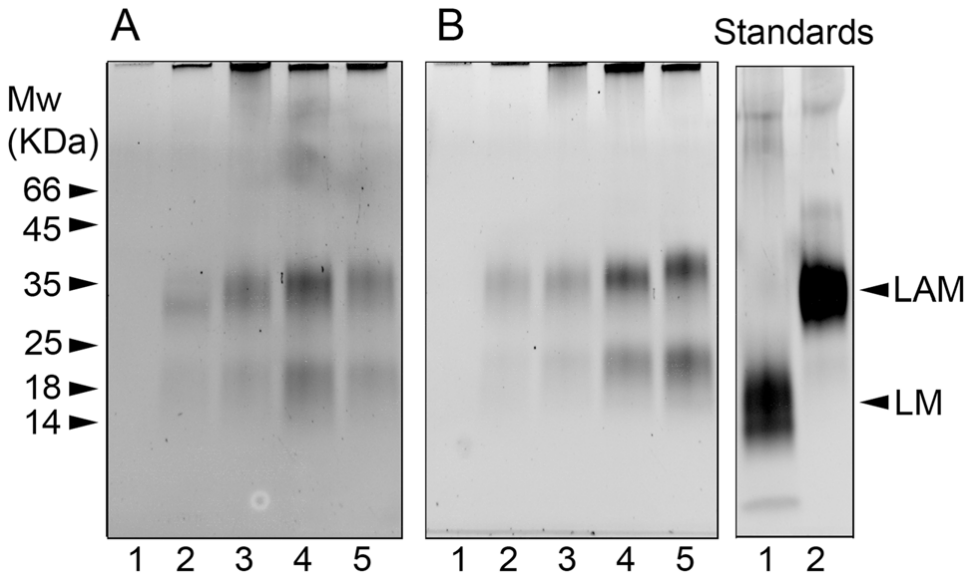
SDS-PAGE analysis of polysaccharide material isolated from the culture medium during the four phases of growth in culture 2 and culture 3. Culture 2 in Panel A; polysaccharide material was purified from liquid medium prior to inoculation in lane 1, exponential phase growth (day 8) in lane 2, stationary phase growth (day 44) in lane 3, late stationary phase growth (day 107) in lane 4 and NRP phase growth (day 292) in lane 5. Culture 3 in Panel B, polysaccharide material was purified from liquid medium prior to inoculation in lane 1, exponential phase growth (day 4) in lane 2, stationary phase growth (day 29) in lane 3, late stationary phase growth (day 72) in lane 4 and NRP phase growth (day 278) lane 5. For comparison, the panel on the right shows LM (lane 1) and LAM (lane 2) previously isolated from *M. tuberculosis* H37Rv cultured exponentially in liquid medium.

Our initial Alcian blue staining of the extracellular material accumulating during growth (Figure 3) directed us to investigate the hypothesis that polysaccharide material derived from *M. tuberculosis* was accumulating in the spent culture medium. Highly purified carbohydrate extracts (representing glycolipid and lipoglycan macromolecules shed from *M. tuberculosis* in liquid medium) were extracted from spent culture medium that had been collected from Culture 2 and Culture 3 in uniform volumes during each of the four phases of growth. Each fraction was subjected to SDS-PAGE analysis and specifically stained to visualise species of lipoglycans and polysaccharides separated by molecular mass (Figure 6) [15]. There was a gradual increase over time in the apparent molecular mass of the band corresponding to lipoarabinomannan (LAM) (Figure 6A and 6B). Interestingly, the position of the lower band, representing lipomannan (LM), remained unchanged over time. This lipoglycan profile correlated with an increase in the molecular mass of LAM (with respect to its initial mass at the start of culture). In order for us to determine the total sugar composition in each of the fractions presented in Figure 4, highly purified lipoglycan material was chemically modified in order to produce alditiol acetate derivatives which were subsequently analysed via gas chromatography (GC) as described previously [16] [15]. Both cultures (2 and 3) displayed a similar phenotype in terms of the overall total sugar content, with a relative increase in the amount of Ara, with respect to Man, over the duration of the two independent culture experiments. An increase in the ratio of Ara: Man was observed as follows: Culture 2, day 8 (1.03), day 44 (3.62), day 107 (4.03) day 292 (5.03) and Culture 3, day 4 (1.21), 29 (3.00), 72 (5.76) and 277 (5.27). Cell wall extracts from Culture 3 were also chemically modified in order to produce alditiol acetate derivatives, which were analysed by GC. An increase in the ratio of Ara: Man was observed as follows: day 4: 0.96, day 29: 0.87, day 72: 1.13 and day 277: 1.63, reflecting the increased Ara:Man ratios also observed in the cell biomass. The ratio of Ara:Man has been shown to have an impact on the immunological properties of LAM [15].

### Global gene expression analysis

Gene expression analyses were applied to understand more about the molecular genetics underlying the transition between each growth phase in culture and particularly to the biochemical changes observed in the cell wall. Whole genome gene expression analyses were performed throughout the time-course for Cultures 1 and 2. Probabilistic analyses using Gaussian process regression and Bayesian model selection were applied to identify genes which showed similar gene expression trends in both cultures [17]. Using the probabilistic model, expression profiles of approximately fifty percent of the genes showed similar dynamics profiles across the time-courses in the two cultures and therefore their profiles could be merged. The remaining genes showed either different gene expression trends in both cultures or the signal present in the data was obscured (in one of the cultures or in both cultures) by the noise level. Details of the probabilistic analysis applied to identify consistent genes between the two cultures are given in (Methods S1) in the supporting information. The merged profiles were arranged in a total of 55 clusters based on the trend in their expression levels, using Bayesian hierarchical clustering of curves [18]. Only those genes that could be merged in terms of their expression profiles in the two cultures were included in the analysis described below. (All 55 cluster profiles can be found at http://xenakis.mrc-bsu.cam.ac.uk/wernisch/enrichment/html/ “An overview of all clusters”). The gene expression data were not normalised to account for the potential reduction of RNA in stationary phase and NRP because there was likely to be a heterogeneous mixture of cells that were in different phenotypic states and therefore an assumption could not be made that all the cells in the stationary and NRP phases were equivalent in their total RNA levels; this could have added further bias and inaccuracies to the data analysis.

**Figure 7:**
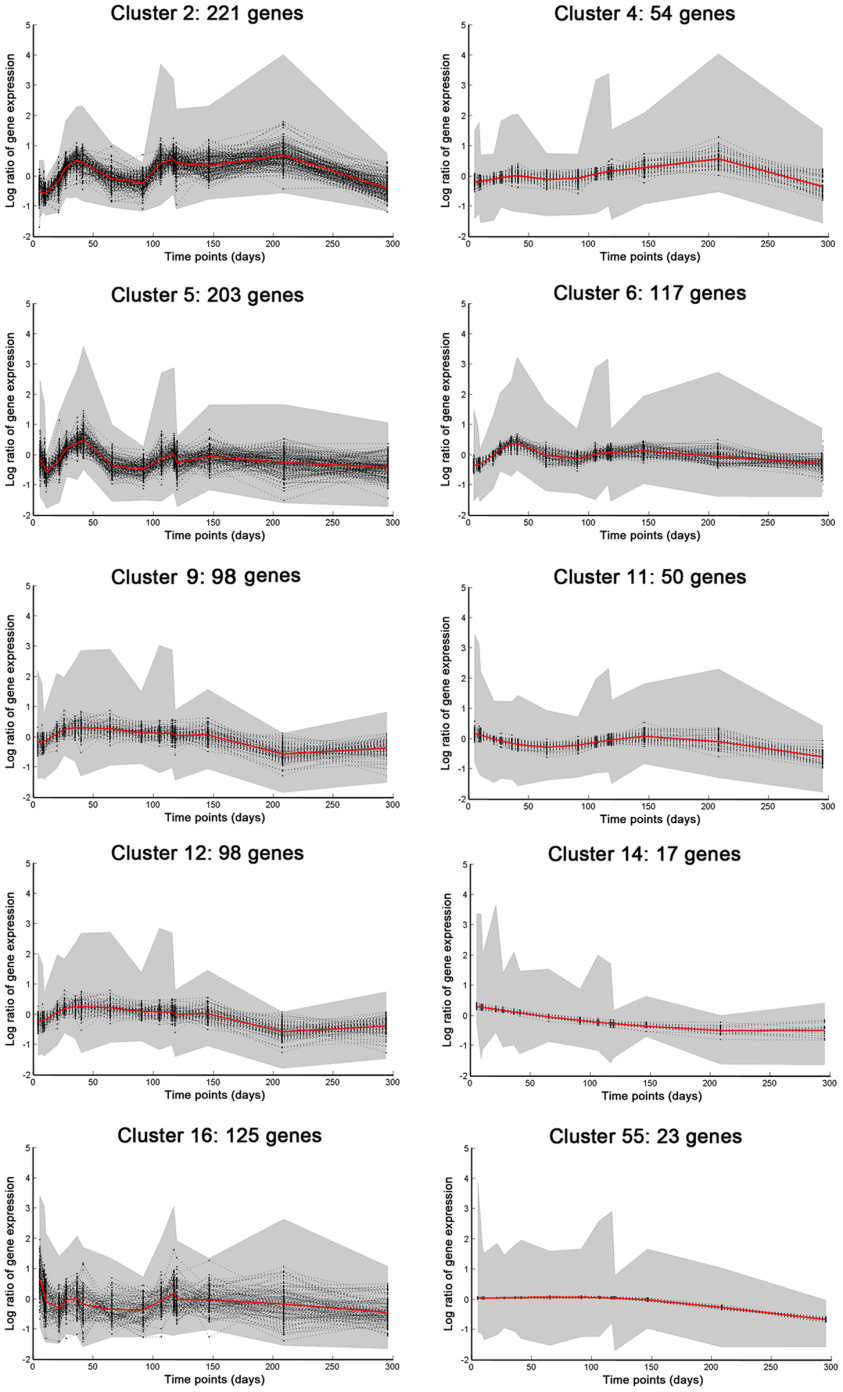
Gene expression clusters with a marked enrichment in fatty acid metabolism, lipid degradation, and cell wall re-modeling. Graphs show normalised log expression values (centered around a mean value of zero) over the time-courses in Cultures 1 and 2. The shaded area represents the 99% confidence interval for the curves.

Most of the 55 clusters were down-regulated in the NRP after day 150 and a huge downward shift in the metabolic response of the bacteria as they enter an NRP state. Eleven clusters containing 561 genes were up-regulated in stationary phase and late stationary phase (compared with exponential phase) followed by down-regulation at approximately day 150 at the start of the NRP phase. Twenty-one clusters containing 315 genes followed a trend of down-regulation from exponential phase right through to the end of the NRP phase. Eight clusters containing 157 genes revealed a profile of flat expression in exponential phase and through stationary phase followed by down-regulation from day 150 in NRP. For eight of the clusters the expression profiles remained flat throughout the time-course. Enrichment for function was applied, using all the gene annotations provide by the Sanger Institute and the MTB-GOA server, to determine which functional groups of genes were important for establishment of an NRP state. The complete results of the enrichment analysis are provided as hyperlinked HTML files as supporting information http://xenakis.mrc-bsu.cam.ac.uk/wernisch/enrichment/html/. “Sanger classification enrichment” and “MTB-GOA classification enrichment”. Some of the gene clusters showed a marked enrichment in fatty acid metabolism, lipid degradation and cell wall re-modeling (Figure 7). Details of the clusters that show enrichment for functional category are presented in Table 1. A list of all the genes that have been enriched for a functional class can also be found at http://xenakis.mrc-bsu.cam.ac.uk/wernisch/enrichment/html/ “Genes of cluster enrichment analysis”.

**Table 1:** Annotation of clusters that were enriched for functional category.

Cluster	Function	Description	P	Ratio	f0-c0	f1-c0	f0-c1	f1-c1
14	I.B.6.a	Aerobic	0	33.7	1883	17	13	4
9	I.H.3	Acyltransferases and Mycolyltransferases	0.91	5.9	1806	13	94	4
55	-	LAM/LM	0.23	13.98	1874	20	20	3
51	II.A.1	Ribosomal protein synthesis, modification	0.48	10.56	1865	33	16	3
16	I.B.8	ATP-proton motive force	0	74.16	1791	1	120	5
16	GO:0050896	Response to stimulus	0.47	5.69	1779	13	120	5
16	GO:0044464	Cell part	0.33	1.99	1547	245	95	30
16	III.B	Chaperones/Heat shock	0.1	14.72	1788	4	121	4
16	II.B.4	Polysaccharides, lipopolysaccharides	0.25	21.91	1790	2	122	3
16	III.D	Protein and peptide secretion	0.14	4.46	1765	27	117	8
16	GO:0032940	Secretion by the cell	0.25	21.91	1790	2	122	3
11	GO:0009072	Aromatic amino acid process	0.39	38.45	1865	2	48	2
11	GO:0042401	Metabolic process	0.39	38.45	1865	2	48	2
11	GO:0003677	DNA binding	0.31	13.12	1858	9	47	3
6	GO:0050662	Coenzyme binding,	0.45	7.91	1792	8	113	4
6	GO:0000166	Nucleotide binding	0.45	7.91	1792	8	113	4
13	GO:0005886	Plasma membrane	0.12	2.57	1266	600	23	28
5	GO:0071767	Mycolic acid metabolic process	3.44	3.3	1701	13	198	5
44	II.A.1	Ribosomal protein synthesis, modification	0.06	12.87	1863	32	18	4
4	I.G.2	Folic acid biosynthesis	0.22	15.51	1856	7	51	3
4	GO:0051234	Establishment of localisation	0.23	70.7	1862	1	52	2
4	GO:0045927	Positive regulation of growth	0.08	12.29	1851	12	50	4
2	IV.C.1.a	PE subfamily	0.16	4.19	1679	17	212	9
2	VI	Unknowns	0.1	1.95	1533	163	183	38
2	GO:0075136	Response to the host	0.38	3.55	1676	20	212	9
46	VI	Unknowns	0.4	5.24	1706	195	10	6
46	GO:0010608 GO:0032268	Post-transcriptional regulation	0.1	66.26	1897	4	14	2
46	GO:0009891	Positive regulation	0.34	29.7	1892	9	14	46

Annotation by the Sanger Institute is indicated by roman literals, GO annotation by the GO label, *p*-values are in percent (not corrected for multiple testing), ratio is the Fisher estimate of the odds ratio and membership of a gene for a cluster (c) or a function (f) is indicated by 1 for membership and 0 for non-members. Lists of genes from the f1-c1 category (joint members of functional class) and are provided in the supporting information at http://xenakis.mrc-bsu.cam.ac.uk/wernisch/enrichment/html/.

**Figure 8:**
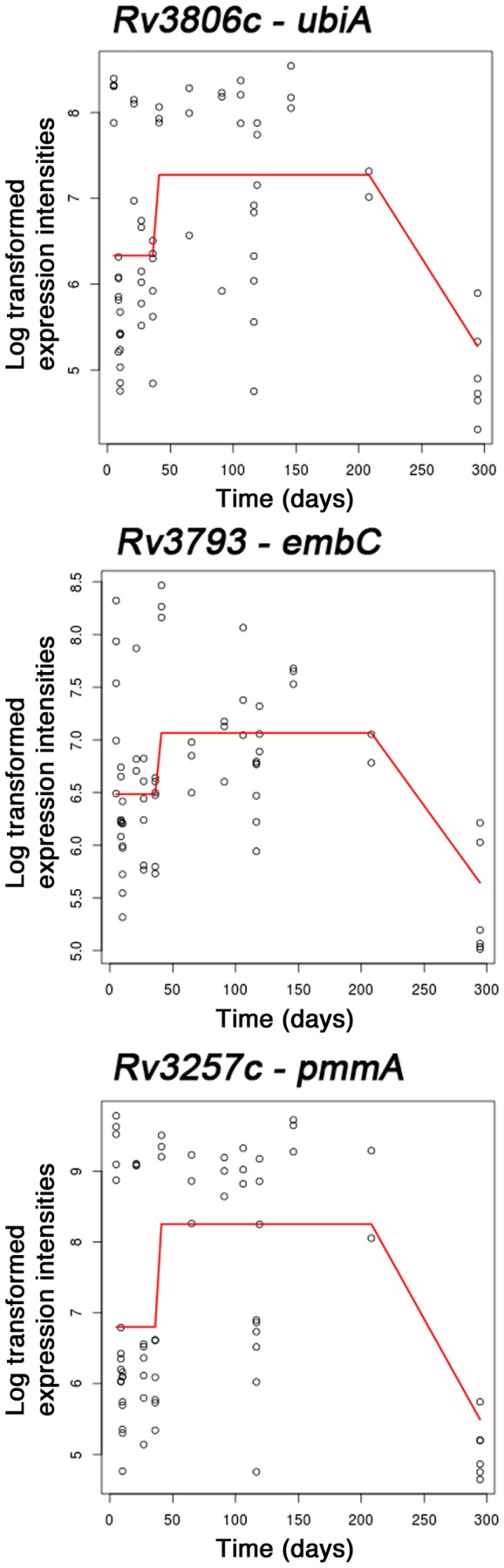
Gene expression profiles for LAM biosynthetic genes, Rv3257c (*pmmA*), Rv3793 (*embC*), Rv3806c (*ubiA*). The lines on the graphs show the average normalised log ratios of gene expression over the time-courses in Cultures 1 and 2.

We have observed changes in the LM, LAM, and free mycolate in relation to an NRP state and so we focused specifically on the genes associated with the biosynthesis of these molecules. We constructed a comprehensive list of genes (Table S2, supporting information) that have been implicated in the PIM→LM→LAM pathway [19] [20]. Using the enrichment analysis method, genes Rv3257c (*pmmA*), Rv3806c (*ubiA*), and Rv3793 (*embC*), were the only genes from the list of LM/LAM biosynthetic genes that were enriched. All three of these genes were found in cluster 55, showing that the dynamics in the expression profiles for these genes were very similar with sustained up-regulation from early stationary phase until day 150 (Figure 8). *PknH* (Rv1266), was revealed to be enriched for in cluster 9 (Figure 7) and evidence suggests that it is directly implicated in the regulation of LAM biosynthesis [21] and was also induced in chronically infected mice (Table 2) [22]. The GO:0071767 annotation for “mycolate acid metabolic process” was used to look for the enrichment of genes involved in the accumulation of free mycolate. Cluster 5 was enriched for genes of the mycolic acid metabolic process and the profile for this cluster consisted of induction early in stationary phase at day 30 followed by further induction at day 116 and day 146. More specifically, genes involved in the synthesis and processing of mycolates were up-regulated early in stationary phase and these were Rv0643 (*mmaA3*, methoxy mycolic acid synthase 3), Rv1273c (transmembrane ABC transporter), Rv1349 (*irtB*, iron-regulated transmembrane ABC transporter), Rv2006 (*otsB1*, trehalose-6-phosphate phosphatase), and Rv3801c (*fadD32*, fatty-acid-AMP synthetase).

**Table 2:** Genes up-regulated in late stationary phase and during chronic infection in mice.

Gene ID (Rv no.)	Gene Name	Cluster no.	Function	Fold up-regulation [22]
Rv2667	*clpX'*	2	ATP-dependent protease ATP-binding subunit	1.92
Rv2853	PE_PGRS	4	PGRS subfamily of gly-rich proteins	2.37
Rv2612c	*pgsA*	16	PIsynthase/CDP-diacylglyceride – inositol phosphatidyltransferase	3.47
Rv1266c	*pknH*	2	Serine/threonine-protein kinase	5.4
Rv2439c	*proB*	4	Glutamate 5-kinase protein	3.55
Rv0612	Rv0612	2	Conserved hypothetical protein	2.2
Rv0666	Rv0666	2	Membrane protein	6.26
Rv2850c	Rv2850c	4	Magnesium-chelatase	3.53
Rv2891	Rv2891	2	Conserved hypothetical protein	3.63

Genes up-regulated in late stationary phase in Cultures 1 and 2 of *M. tuberculosis* and at 60 days post-infection in the BALB/C mice [22]. The cluster profiles for the genes listed can be found in the supporting data at http://xenakis.mrc-bsu.cam.ac.uk/wernisch/enrichment/html/.

### Adaptation of central metabolism

Gene expression profiling was also performed to investigate energy metabolism in *M. tuberculosis* during its transition into an NRP state and how the cells maintained their energy levels in NRP. Previous studies have observed that nutrient-starved, non-replicating bacilli undergo a global down-regulation of metabolic genes involved in respiration [23] [10] [24]. Genes involved in the degradation of fatty acids via the β-oxidation pathway (Rv0914c, keto acyl-CoA thiolase) were up-regulated early around day 41 in Cluster 9 (Figure 7) and showed sustained up-regulation until about day 150. Cluster 16 (Figure 7) is one of the largest clusters containing 125 genes and had several time-points at which the induction of gene expression occurred in exponential phase, stationary phase and late stationary phase, followed by down-regulation throughout NRP. Cluster 16 was enriched for in several Sanger and GO functional groups and contained genes involved in energy metabolism such as ATP synthases (Rv1305, Rv1306, Rv1308, Rv1309, and Rv1310). Cluster 16 is generally enriched in genes for biosynthesis of lipids such as Rv0242c (*fabG4*), Rv0673 (*echA4*), Rv0271c (*FadE6*), or Rv0672 (*FadE8*), or lipopolysaccharides and phospholipids (Rv3032, Rv0062 (celA1), and Rv0315). In Cluster 2 (221 genes) (Figure 7), the induction of gene expression occurred at around day 41; following this was a reduction in gene expression with a further induction at day 208. Genes included in this cluster were Rv1169c (*lipX*) and Rv0467 (*icl1*), which confirmed that the induction of the β-oxidation of fatty acids and lipid degradation are important for the survival of *M. tuberculosis* on diminishing levels of fatty acid. Cluster 6 (Figure 7), showed early induction at day 41 with a second peak of expression at day 146, and comprised genes coding for nucleotide binding proteins involved in arginine biosynthesis Rv1652 (*argC*) and NADPH requiring oxidoreductases (Rv3106 (*fprA*), Rv3303 (*lpdA*)) involved in energy metabolism. Genes involved in the regulation of lipid metabolism, (Rv3574 (*kstR*) [25] were enriched for in Cluster 11 (Figure 7), which showed late gene induction at day 146 followed by down-regulation thereafter. Furthermore, genes involved in the biosynthesis of aromatic amino acids also fell into this cluster (Figure 7). We were surprised to see a few clusters with genes induced in NRP at around day 208. Cluster 4 (Figure 7) contained folic acid biosynthetic genes, Rv3607c (*folB*), Rv2447c (*folC*) and Rv3608c (*folP1*), which were up-regulated at this late stage. It also contained genes involved in transport and ion channels, Rv3065 (*mmr*) and Rv0985c (*mscL*), as well as the toxin-antitoxin *vapBC* family under the GO term for positive regulation of growth. Cluster 14 (Figure 7) contains genes that were down-regulated throughout the time-course. The profiles of genes which are involved in energy metabolism, in particular, NADH dehydrogenases (Rv3156, Rv3158, Rv3154, and Rv2194), indicate a reduction in aerobic respiration from exponential phase. Two acyltransferases Rv2482c (*plsB2*) and Rv2881 (*cdsA*) were also enriched for in this cluster (Figure 7).

### Comparisons with other gene expression studies

Comparisons were made between genes that were more highly expressed in stationary phase/late stationary phase (than in the exponential phase, 561 genes, (Table S3, supporting information). in our nutrient-starved cultures and the genes induced in previously described *in vivo* models of *M. tuberculosis* infection and *in vitro* models of nutrient-starvation [22] [26] [27] [23] [10] Table 2 displays genes that were induced in stationary phase in our cultures and at 60 days post-infection in chronically infected mice [22]. One fifth of the stationary phase-induced genes from our cultures were induced at 48 hours post-infection in the macrophage (Table S3, supporting information) [26]. Of these genes, nine genes were found to be induced in at least one of the other *in vivo* or *in vitro* data sets used for this comparison (Table 3). Genes Rv3555c, Rv2642, Rv0083 were induced in the human granuloma [27] and gene Rv0251c, encoding heat shock protein *hsp*, was the only gene induced under all the conditions compared.

**Table 3:** Genes up-regulated in stationary phase in Cultures 1 and 2 and in further conditions *in vitro* or *in vivo*.

Gene ID (Rv no.)	Gene Name	Clusterno.	Function	Also up-regulated in the following studies:
Rv0083		2	Oxidoreductase	Human granuloma v *in vitro* culture in 7H9; [27]
Rv0122		2	Hypothetical protein	96h starvation in PBS [23]
Rv0251c	*hsp*	16	Heat shock protein	Human granuloma v *in vitro* [27] 96h starvation in PBS [23] 75 days starvation [10]
Rv0284		50	Conserved membrane protein	75 days starvation [10]
Rv1072		11	Conserved membrane protein	75 days starvation [10]
Rv1285	*cysD*	2	Sulfate adenylyltransferase	75 days starvation [10]
Rv1461		15	Conserved hypothetical protein	75 days starvation [10]
Rv1462		3	Conserved hypothetical protein	75 days starvation [10]
Rv2497c	*pdhA*	2	Pyruvate dehydrogenase	75 days starvation [10]
Rv2642		14	Transcriptional regulator, arsR	Human granuloma v in vitro culture in 7H9 [27]
Rv2710	*sigB*	16	RNA polymerase sigma factor	75 days starvation [10]
Rv3139	fadE24	3	Acyl-CoA dehydrogenase	75 days starvation [10]
Rv3173c		4	Transcriptional regulator, tetR acrR-family	75 days starvation [10]
Rv3555c		3	Conserved hypothetical protein	Human granuloma v *in vitro* culture in 7H9, Human granuloma v pericavity [27]

**Table 3** Genes that were up-regulated at 48 h in macrophages [26] and in Cultures 1 and 2 of *M. tuberculosis* in late stationary phase (Table S3, supporting information), and in at least one other condition either *in vitro* or *in vivo* model as indicated in Table 3. The details of cluster profiles can be found in the supporting information at http://xenakis.mrc-bsu.cam.ac.uk/wernisch/enrichment/html/.

### Infectivity of M. tuberculosis in the guinea pig

**Figure 9:**
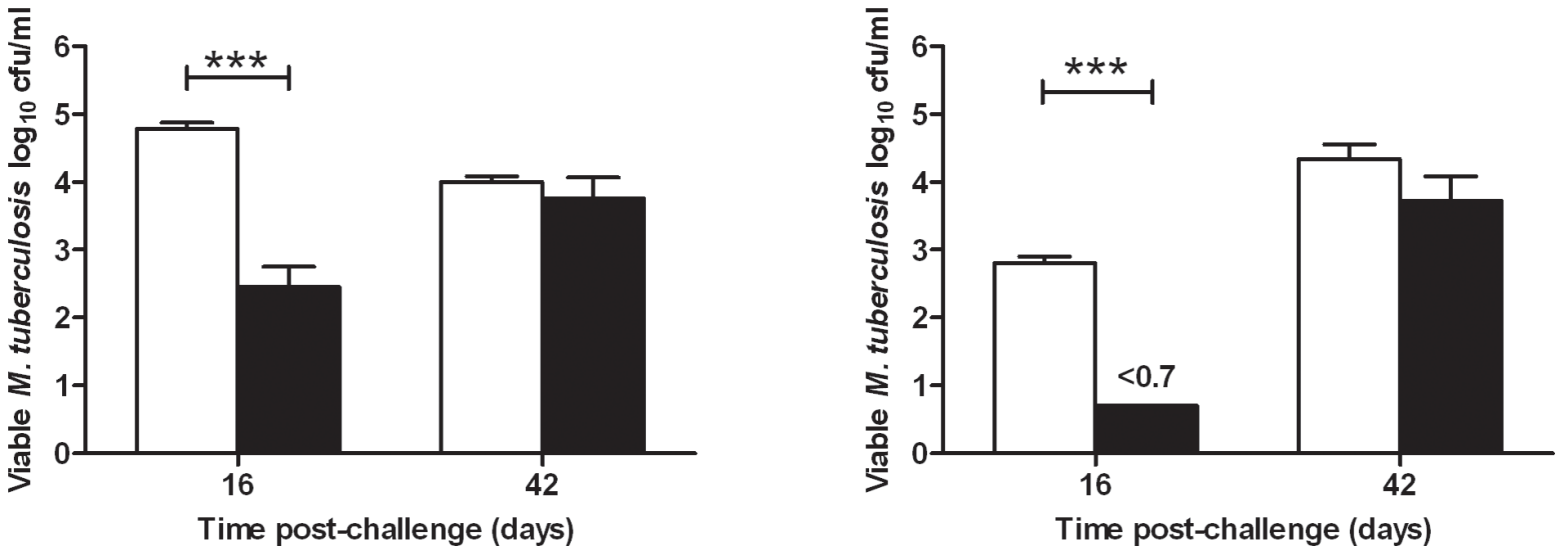
Bacterial load at day 16 and day 42 in guinea pigs post aerosol challenge with either log phase culture or NRP culture. Lungs in Panel A) and spleen in Panel B), challenged with either exponentially growing culture (white) or NRP culture of *M. tuberculosis* (black). Bars indicate group mean log_10_ cfu mL^−1^ +/− S.E.M of 8 guinea pigs. Statistical analysis was performed using paired T-tests. *** indicates *P* = <0.001.

**Figure 10:**
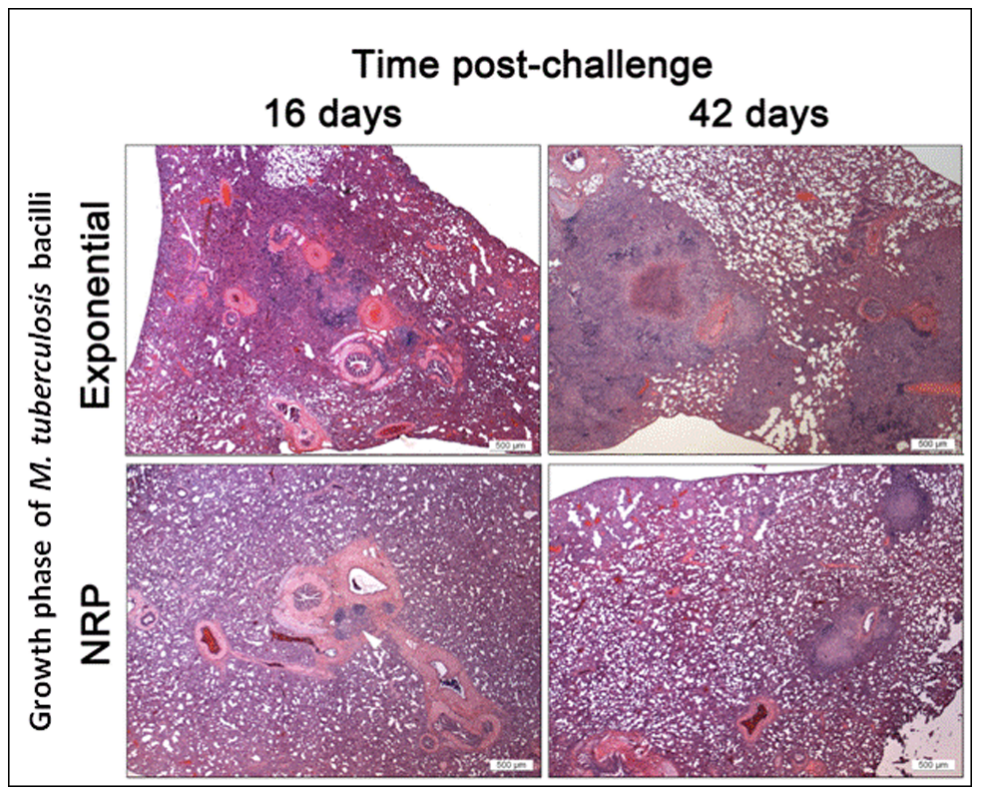
Histopathological changes in guinea pig lung following aerosol challenge with *M. tuberculosis* cultured from different growth phases of nutrient starvation at days 16 and 42 post challenge. H&E. Magnification bar, 500 µm.

**Figure 11:**
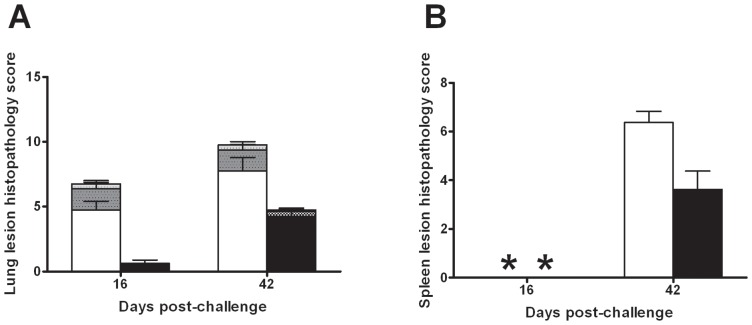
Subjective histopathology scores of *post mortem* at 16 and 42 days post aerosol challenge. Lung in Panel A) and spleen in Panel B) challenged with either exponentially growing culture (white) or NRP cultures of *M. tuberculosis* (black). Bars indicate group mean of 8 animals. Histopathological changes in the lung were recorded either as consolidated (no-shaded bars), necrotic (heavy-shaded bars) or calcified (light shaded bars). Spleen scores for each animal comprise number, size, and foci of necrosis and calcification.

A guinea pig study was performed to determine whether the organism's ability to infect was improved or impaired by the different physiological states we have observed. Bacteria were removed at day 4 (exponential phase), and day 277 (NRP phase) from Culture 3 and used to infect guinea pigs by the aerosol route. In order to achieve a low-dose infection (less than 20 bacilli implanted in the lung), a concentration of approximately 1×10^5^ cfu mL^−1^ was needed in the aerosol generator. The culture samples were adjusted to this concentration using an estimation based on OD which was retrospectively verified by plating on solid agar. The concentration of *M. tuberculosis* in each of the nebuliser solutions was 9.33×10^4^ cfu mL^−1^ (exponential phase) and 2.09×10^4^ cfu mL^−1^ (NRP). Verification of the very low number of organisms implanted in the lungs was not feasible due to the inability to enumerate small numbers of cells in a large sample of lung tissue. However, historical data enabled a relationship between the nebuliser concentration and the delivered dose to be established [28] [29]. On this basis the animals infected with the exponential phase and NRP bacteria received similar doses. The bacterial load (Figure 9) and histopathological changes (Figure 10 and Figure 11) resulting from these infections were determined in lungs and spleens at days 16 and 42 post-challenge.

At day 16 post-challenge, the bacterial load in the lungs and spleens of animals infected with the exponential phase bacteria was significantly higher than in the organs of animals infected with NRP (*P* = <0.001) (Figure 9). Bacteria were not detected (limit of detection  = 0.7 log_10_ cfu mL^−1^, Figure 9) in the spleens of NRP-challenged animals. By day 42 post-challenge, the lung bacterial load had increased (relative to day 16) in animals infected with NRP bacteria, in contrast to the animals infected with exponential phase bacteria where the mean value was lower at day 42 compared to day 16. Statistical analysis showed no significant differences between the mean values of cfu in lungs at day 42 between both groups of guinea pigs (P = 0.471). In the spleens, the bacterial load had increased in both groups of animals relative to day 16 but the increase was more pronounced in the NRP group. The mean values for cfu in the spleen at day 42 were not significantly different from each other by statistical analysis (P = 0.163).

Lesions in the lung and spleen consisted of variable sized granulomatous foci with mainly macrophages and lymphoid cells. Occasional foci of necrosis and calcification were observed (Figure 10). The subjective histopathology scores are summarised in Figure 11; a mean score of all animals in the group is given in the text below and the range is shown in curly brackets. In all animals challenged with exponential phase cells, at day 16, the lungs exhibited histopathological changes with a score of 6.7 {2–13}. In comparison, there was much reduced pathology in the lungs of the animals challenged with NRP phase bacteria (score  = 0.6 {0–2}) and 4 out of 8 animals had no lesions. At 42 days post-challenge, there was more extensive lung pathology (than at day 16) in both of the groups but the lowest pathology was observed in animals challenged with the NRP phase bacteria (4.75; {3–9}). This level of pathology at day 42 was similar to that observed in the animals infected with exponential phase at day 16 post challenge. Histopathological changes were not observed in the spleen in any animal at 16 days post-challenge. At day 42, the pathology in animals infected with NRP bacteria (3.6 {0–7}) was lower than exponential phase (6.4 {5–9}) infected animals.

## Discussion

### NRP bacilli can be generated by extended nutrient-depletion

The effects of nutrient-depletion on the persistence and survival of *M. tuberculosis* were assessed independently of the effects of low oxygen in employing controlled batch cultures [30]. An extended period of nutrient-depletion resulted in a population of bacteria that were culturable on agar and maintained a consistent viable count; a population that we have termed NRP. We recognise that during this phase, the cultures were likely to contain a heterogeneous mixture of organisms, some of which were non-replicating, whilst other bacilli could be dividing or viable but not culturable, thus reflecting the heterogeneity of cell states within the granuloma [31]. This study involved growing *M. tuberculosis* in a medium that contained oleic acid (derived from Tween 80), as the primary carbon source, which reflects the nutrient sources available *in vivo* [4]. We have previously shown that *M. tuberculosis* and *Mycobacterium bovis* will utilise Tween 80 in the absence of glycerol and glucose in continuous culture [11]. Tween 80 is cleaved to liberate oleic acid and a polyethylene derivative of sorbitol; each component is then subsequently absorbed and metabolised through the glyoxylate shunt and fatty acid degradation biochemical pathways [32]. Eighty percent of the available Tween 80 was being metabolised by our cultures in the current study (assuming that one molecule of Tween 80 was hydrolysed to one molecule of oleic acid) (Figure S1, supporting information). It seems unlikely that an NRP phase would be established if free fatty acids were still available. However, the assay used (Free fatty acid half micro kit, Roche) is a non-discriminate measure of different free fatty acids levels; oleic acids in the supernatant could have been further hydrolysed to alternative fatty acids that were not available to *M. tuberculosis* as a carbon source.

The current paradigm concerning non-replicating, dormant and persistent sub-populations of *M. tuberculosis* relies heavily upon the notion that adaptation of the bacilli to anaerobiosis is key to maintaining cell viability for a prolonged period of time, which is mediated by the DosR-regulated dormancy regulon [33]. Our previous findings using steady-state chemostat cultures show that the DosR regulon is also up-regulated in actively dividing bacilli (at a doubling time of 23 h) growing in very low levels of oxygen (0.2% dissolved oxygen tension) [30]. These data highlight that there are likely to be environmental cues *in vivo* in addition to hypoxia that are encountered by *M. tuberculosis* in the granuloma, such as nutrient-limitation and alternative carbon sources [4]. However, there are a limited number of *in vitro* studies that have investigated nutrient-depletion as a potential stimulus for triggering the transition of *M. tuberculosis* into an NRP state. Loebel *et al*. [34] investigated the effect of nutrients predicted to be available in a granuloma on the metabolism of *M. tuberculosis* by transferring cells from nutrient-rich medium into phosphate-buffered saline (PBS) and measuring the respiration rate. Nutrient-starvation resulted in a gradual shutdown of respiration to minimal levels, but bacilli remained viable and were later able to recover on rich medium. Betts et al. (2002) used a similar approach of starvation in PBS in static, sealed bottles to observe the effects of nutrient depletion and hypoxia. This study, and other *in vitro* models of dormancy, combined with post-genomic approaches, have provided further evidence of Loebel's early work by showing that nutrient-starved, non-replicating bacilli undergo a global down-regulation of metabolic genes involved in respiration [23] [10] [24].

Lower expression levels of genes involved in ATP synthesis were observed in our NRP cultures whereas there were increases in the expression of genes such as *icl1* (isocitrate lyase) and genes associated with the β-oxidation of fatty acids. This response was also reflected in the nutrient-starved cultures described in this study, since *icl1* and other genes involved in the degradation of fatty acids *via* the β-oxidation pathway were induced (Rv0914c, keto acyl-CoA thiolase). The induction of *icl1* is an important observation as it is known to be essential for *M. tuberculosis* to persist in mouse macrophages [35] and has more recently been shown to be important for utilisation of cytotoxic propionyl-CoA and its conversion to succinate [36]. These observations and the finding that a large proportion of genes induced in stationary phase were also induced in the macrophage (Schnappinger *et al.* 2003) (Table S3, supporting information) provide supporting evidence that adaptation to nutrient-limitation and the metabolism of fatty acids as a carbon source are important *in vivo*.

Folic acid biosynthetic genes, Rv3607c (*folB*), Rv2447c (*folC*) and Rv3608c (*folP1*), were up-regulated throughout late stationary phase and into the NRP phase, only to drop in their expression level at the end of the NRP phase. Intermediates from the folic acid biosynthetic pathway are known to be incorporated into the molybdopterin biosynthetic pathway [37], and molybdopterin synthesising enzymes have been shown to catalyse important redox reactions during dormancy regulation, the metabolism of energy sources, and nitrogen sources. The importance of molybdopterin biosynthesis as a cofactor in *M. tuberculosis* is a relatively unexplored area, but warrants further investigation [38].

### The composition of extracellular material

Previously, it has been demonstrated that an increased production of an amorphous material composed of protein and polysaccharides, arabinomannan and glucan followed the growth curve of *M. tuberculosis*, [39] [40] [41]. We also observed a similar appearance of an extracellular material which accumulated over the duration of the experiment (Figure 2). A gradual accumulation of free mycolates in the cell wall lipids was clearly observed, which was accompanied by a concomitant reduction in the levels of TDM and TMM (Figure 4). The induction of a number of mycolate biosynthetic and processing genes in early stationary phase supports the finding that free mycolates gradually accumulated in culture, particularly Rv2006 (*otsB1*), which is involved in the final steps of mycolic acid biosynthesis and serves to activate meromycolic acid into meromycolyl-AMP thereby transferring the meromycolic acyl chains onto *pks13* [42]. Ojha *et al.* [43] previously showed that free mycolates played a key role in the formation of mycobacterial pellicle biofilms and these authors also demonstrated that TDM is directly cleaved, thus liberating free mycolate [44]. However, until now, the environmental factors that stimulate *M. tuberculosis* to remodel its cell wall and induce production of free mycolate have not been investigated fully. Conventionally, biofilm formation starts with microbial attachment to a surface [45]. However, freely dispersed aggregates or “flocs” of bacteria have been described for other bacterial pathogens, similar to the aggregates of bacilli observed in our cultures [46]. *M. tuberculosis* biofilms, *in vivo*, have been described by Canetti who observed dense sheets of bacilli, that were not adhered to a surface, within the caseum of the granuloma [47].

We observed an increase over time not only in the quantity of LM and LAM, but also in the size and arabinose/mannose ratio of the LAM (Figure 6). It appeared that *M. tuberculosis* was altering the structure of LAM by increasing the level of arabinosylation of the mannan domain in response to nutrient-depletion. This is in contrast to the findings for *Mycobacterium smegmatis*, in which Dhiman *et al.* [48] showed that the Ara to Man ratio decreased in the cell biomass over time. This is the first time that LAM has been shown to be associated with adaptation of *M. tuberculosis* to nutrient-depletion *in vitro.* This finding is also supported by the transcriptomic analyses showing that several genes involved in the key stages of LAM biosynthesis such as Rv3257c (*pmmA*), Rv3806c (*ubiA*), and Rv3793 (*embC*) are up-regulated in early stationary phase and through late stationary phase (Figure 8). Gene *ubiA* encodes for decaprenyl-phosphate 5 phospho-ribosyltransferase, which is required to produce a key intermediate leading to the biosynthesis of decaprenylmonophosphoarabinose (DPA) [49], which is the sole substrate utilised by the membrane bound arabinosyltransferases (AraTs) in the formation of D-arabinan in mycobacteria [50]. In this regard, EmbC is an α(1→5)-arabinofuranosyltransferase which serves to elongate the 5-Ara*f* linkages of the arabinan domain of LAM [15]. The function of EmbC is specific to LAM biosynthesis and is regulated by the action of PknH [51]. Our microarray data indicate that *pknH* is up-regulated in stationary phase (Cluster 9, Figure 7) and is the cognate kinase that phosphorylates EmbR, which in turn regulates *embC* expression [52]. It is therefore plausible to speculate that PknH senses nutrient-depletion as a stimulus by an as yet unknown mechanism. The resulting signal is then transduced via the Ser/Thr kinase response network into a response which increases the expression of *embC* thereby increasing the arabinose content of LAM. Apart from the increase in free mycolates and LAM/LM ratio observed in culture, no other changes in any of the other lipids (including triacylglycerols (TAG)) or carbohydrates could be observed. Previously reported dormancy models have shown an apparent increase in TAG, but these have been under alternative growth conditions, which serve to highlight the contributions made by different microenvironments with respect to non-replicating persistence [53] [54] [55] [56].

We have previously shown that a deletion in *aftC* (an α(1→3) arabinofuranosyltransferase) resulted in the truncation of the arabinan domain of LAM, which drastically altered the immunological properties of this truncated molecule (termed AftC-LAM) making it more pro-inflammatory in comparison to “wild type” LAM [15]. This key difference has been attributed to the way in which the arabinan domains of AftC-LAM have a reduced effect of “masking” the pro-inflammatory nature of the mannose core of the LAM molecule, thereby altering its immunogenic properties. More recent evidence showed that AftE is involved in the biosynthesis of single arabinans of LAM. Deletion of the *aftE* gene resulted in hyper-mannosylated LAM, which is a stronger inducer of cytokine production *in vitro* than LAM [57]. We observed a clear increase in the expression of *embC*, which is likely to be responsible for the observed increased arabinosylation of LAM over the time-course (Figure 8). Therefore, it is plausible that the LAM molecules matured towards the end of culture period, particularly during NRP and have increased the “masking” of the mannan domain, which in turn is likely to exert a transient effect on the pathogenicity of *M. tuberculosis in vivo*, due to the altered immunogenic properties of the lipoglycans being produced. The mycolic acid layer provides a hydrophobic mesh-like structure for the intercalation of additional complex lipids and lipoglycans; therefore, we hypothesise that stationary phase and NRP phase bacilli are accumulating additional quantities of free mycolate through increased cleavage of TDM to serve as a scaffold for the production and export of LM and LAM into the extracellular matrix. TDM is required for a pro-inflammatory response and the formation of granulomas via the mincle pathway [58] [4]; the proportions of cell-associated TDM/TMM/free mycolate, combined with the altered inflammatory properties of hyper-arabinosylated LAM, could be having an important impact on the outcome of the early stages of infection. These findings might also have wider reaching implications as EmbC is the target of the front-line drug ethambutol [59].

### Effect of nutrient depletion on pathogenicity

NRP bacilli were able to successfully infect guinea pigs. We compared the pathogenicity of bacilli from exponential and NRP phases in guinea pigs when delivered via the aerosol route, using bacterial replication and organ pathology as measures of infectivity. Bacilli from the NRP phase were different in each of these parameters compared to the exponential phase bacilli. In the early stages of infection, NRP infected animals showed a significantly lower bacterial load than the exponential phase infected animals and minimal pathological changes were observed. However, by day 42 post-challenge, the bacterial load in the NRP group was similar to the animals infected with exponential phase bacilli. In contrast, the pathology observed in the NRP infected animals at the later time point, although increased compared to day 16, did not reach equivalent levels to that caused by the exponential phase bacteria. Indeed, the extent and characteristics of the pathology in the NRP-infected animals was very similar to that observed in the exponential phase group but at the earlier time-point. The usual course of infection following low-dose aerosol challenge of guinea pigs is an initial replication in the lungs with a peak in the bacterial load at around 3–4 weeks, followed by a slight decrease to a level which is then sustained for a prolonged period of 15–20 weeks [60]. This control of bacterial replication is mediated by an on-going immune response which results in a steady increase in pathological features such as cellular infiltration and granuloma formation. The animals infected with exponential phase bacteria demonstrated this pattern whereby the bacterial load had stabilised by day 42 but the pathology continued to increase. In contrast, the observations in the animals infected with the NRP bacilli were consistent with a delay in the development of disease; at day 16 post-infection the bacteria were in the early stages of replication and there was little or no immunopathology observed. By day 42, the bacterial load had reached a plateau that was at a similar level to those animals that were infected with exponential phase organisms. However, at this time-point the pathological features were consistent with a less advanced stage of immune interaction. We propose that the NRP cells remained in a non-replicating state for a period of possibly up to 2 weeks after aerosol challenge. Adaptation to the *in vivo* environment allowed reversion to a phenotype which resulted in progressive infection and disease. The host and bacterial changes which trigger this replication may be similar to those which occur during reactivation of latent infection in humans. The only other published study reporting on the virulence properties of NRP cells utilised a ‘Wayne-type’ hypoxia to generate NRP bacilli which were used to infect mice via the intranasal route [61]. Despite many differences between this study and ours, there was a similarity in the finding that the bacterial load was lower in the early stages post-inoculation when compared to “regular” cultures. Therefore, whether induced by hypoxia or nutrient starvation, it appears that NRP bacteria retain their capacity to establish an infection in the susceptible host but with a reduced infectivity.

### Concluding remarks

In comparison to exponentially growing cells, NRP bacteria exhibit reduced infectivity for guinea pigs, which coincides with significant alterations to the cell wall components known to be associated with host-pathogen interactions. Gene expression analysis of the biochemical pathways leading to the assembly of these important molecules lends additional support to our hypothesis that, upon prolonged nutrient limitation, NRP bacilli exhibit a drastically altered cell wall phenotype which corresponds with a reduced infectivity profile. It could be that NRP phase organisms were unable to initiate replication and interaction with the immune system due in part to alterations in the composition of free mycolate and LAM. This now warrants further investigation as physiological changes such as these could have a key role in the establishment of latent disease. Similarly, the changes which occur in both the bacteria and the host as replication re-establishes will provide insights to the mechanisms associated with reactivation of tuberculosis disease.

## Materials and Methods

### Ethics Statement

The studies were conducted according to UK Home Office Legislation for animal experimentation and were approved by a local ethical committee at the Health Protection Agency, Porton Down, UK. The project licence number under which the work was completed was PPL 30/2704.

### Strains and medium

Studies were performed with *M. tuberculosis* strain H37Rv (NCTC cat. no. 7416). Stock cultures were grown on Middlebrook 7H10+ OADC for 3 weeks at 37±2°C.

### In vitro model of mycobacterial non-replicating persistence under nutrient-starved conditions

Cultures were established in CAMR Mycobacterium Medium Mod 6 (CMM Mod 6) [12]. The first two components of the medium were added to the first volume of water. The remaining components were added in the order listed (Table S1, supporting information). The pH was adjusted to 6.5 using 20% potassium hydroxide solution (w/v in distilled water). The medium was filter sterilised by passage through a 0.1 μm pore size cellulose acetate membrane filter capsule (Sartorius Ltd). Middlebrook 7H10+ OADC agar was used to prepare colonies for inoculation of the cultures and for enumeration of viable bacteria in the cultures.

### Inoculation and culture of M. tuberculosis

The cultures were established following a modification of the method described previously [10]. Culture experiments were performed in a two litre glass vessel operated at a working volume of 1800 mL. The culture was agitated by a magnetic bar placed in the culture vessel coupled to a magnetic stirrer positioned beneath the vessel. Culture conditions were continuously monitored by an Anglicon Microlab Fermentation System (Brighton Systems, Newhaven), linked to sensor probes inserted into the culture through sealed ports in the top plate. The vessel was filled with 1800 ml of sterile culture medium (CMM Mod6) and parameters were allowed to stabilise at 37°C±2°C, pH 6.9±0.3 and a dissolved oxygen tension of approximately 50% air saturation (10% DOT). A dense inoculum was prepared by re-suspending colonies from 5 Middlebrook agar cultures (grown at 37°C±2°C for 3 weeks) in sterile deionised water. The inoculum was aseptically transferred to the culture vessel, to provide an initial culture turbidity of approximately 0.25 at 540_nm_. The culture temperature was monitored by an Anglicon temperature probe, and maintained at 37°C by a heating pad positioned beneath the culture vessel. The culture was stirred at an agitation rate of 500 to 750 rpm. The oxygen concentration was monitored with a galvanic oxygen electrode (Uniprobe, Cardiff) and the air saturation was maintained at 50% (10% dissolved oxygen tension). The initial culture pH was set at 6.7 and was monitored through-out the experiment using an Ingold pH electrode (Mettler-Toledo, Leicester). Each culture was maintained for at least 200 days and samples were removed regularly to monitor growth and survival and for lipid/carbohydrate analysis, infectivity studies and gene expression.

### Growth and survival

Bacterial growth and survival was assessed by determining the number of viable cells in the culture system at specific time-points selected in each phase of growth (exponential, stationary phase, late stationary phase, and NRP phase; Figure 1). This was achieved by preparing a decimal dilution series of the sample in sterile water and plating 100 µL aliquots onto Middlebrook 7H10+ OADC plates in triplicate. The plates were incubated at 37°C for up to 4 weeks before enumerating the number of colonies formed.

### Analysis of Tween 80 levels in spent culture supernatant

Samples of spent supernatant and a sample of the starting medium were hydrolysed to free fatty acids by heating 0.5 mL of sample with 0.5 mL of methanol and 0.2 mL of 25% potassium hydroxide (w/v in water), in an eppendorf tube, to 100°C for an hour. The pH of each sample was adjusted to pH 7.0 (using concentrated hydrochloric acid and 25% potassium hydroxide) prior to fatty acid determination, which was then performed using the “Free fatty acids, Half-micro test” by following the manufacturer's instructions (supplied by Roche, Welwyn Garden City, UK) [62].

### EM analysis

Culture samples (5 mL volumes) were fixed in 4% formaldehyde for at least 16 hours. Fixed cells were immobilised by allowing them to settle onto a poly-l-lysine coated 10 mm glass cover slip overnight in a moist chamber. Immobilised cells were further fixed in 1% v/v osmium tetroxide for 1–2 hours at room temperature. Samples were dehydrated at room temperature through a graded ethanol series from 25% ethanol (v/v) to 100% ethanol (v/v) in steps of 25%. Each dehydration step was performed for 15 minutes. Cover slips were then washed twice in hexamethyldilsilazane (HMDS) for 15 minutes and air dried. The cover slip was mounted onto an SEM stub and a conductive gold coating was applied using an Atom Tech ion beam Z705 ultra fine grain coating unit (approx 10 nm thickness). Specimens were examined using a FEI XL30FEG scanning electron microscope at an accelerating voltage of 4 kV and a working distance of 10 mm.

### Staining for exopolymer production

A few drops of the culture sample were added to silanised slides, spread over the middle of the slide and left to dry overnight in a damp chamber. The slides were pre-treated with 100% (v/v) industrial methylated spirits (IMS) by rinsing the slide for a few minutes in the alcohol and then rinsing with distilled water before the staining method was undertaken. The slides were stained with carbol fuchsin for 10 minutes, rinsed in tap water and de-colorised with 1% hydrochloric acid in 70% IMS (v/v). The slides were then rinsed in tap water and then rinsed in distilled water. The alcian blue solution was applied for 5 minutes at a pH of 1.0, to stain for polysaccharides. Slides were rinsed with distilled water followed by a rinse in 100% IMS (v/v), cleared in xylene, and mounted for light microscopy. Slides were visualised and photographed at a magnification of ×100.

### Extraction of polar and apolar lipids

Polar and apolar lipids were extracted from cell pastes (adjusted to approximately 2.5×10^7^ cells, in order to normalise our analysis) from each culture time-point as described previously [14]. Apolar lipids were re-suspendend in 50 µL of 2∶1 CHCl_3_/MeOH and equivalent volumes were subjected to 1D-TLC with silica gel plates (5734 silica gel 60F_254_; Merck, Darmstadt, Germany), developed in CHCl_3_/MeOH/H_2_O (60∶16∶2, v/v/v) [14]. Equivalent volumes of apolar lipids were also subject to 2D-TLC analysis developed in CHCl_3_/MeOH (96∶4, v/v) for direction 1 and toluene/acetone (82∶2, v/v) for direction 2 [14]. TLC plates were sprayed with 5% ethanolic molybdophoshoric acid (MPA) and charred to visualise the lipids.

### Extraction of lipoglycans from culture supernatant

In order to extract polysaccharide material from a normalised culture supernatant, 50 mL of supernatant fractions collected at each time-point from Culture 2 (d0, d4, d29, d72, and d277) and Culture 3 (d0, d8, d44, d107, d292) were spun down by centrifugation at 3000×g to remove the cells. This step was repeated twice. The supernatants were then stored at −80°C until the end of the culture time-course. The frozen supernatant samples were irradiated for two hours, in a Gammacell 220 instrument, using gamma rays at an energy level of 1.33 MeV. The samples remained frozen throughout the procedure. The supernatants were subsequently defrosted and added to 200 mL of ice cold acetone and stored overnight at −80°C. The resulting precipitate was collected by centrifugation at 5,000*×g* for 1 hr at 4°C. The pelleted material was subjected to treatment with 6 mL of 90% phenol heated to 80°C for 2 hr. After cooling, each sample was centrifuged at 3,500*×g* for 30 min to induce a bi-phase. The upper layer (∼3 mL) was recovered and a further 3 mL of PBS was added to the lower phenol layer, mixed and recovered by centrifugation at 3,500*×g* for 30 min. The upper layer (∼3 mL) was pooled with the previously collected upper layer giving a total sample of 6 mL. After extensive dialysis against water using a MWCO 3,500 dialysis membrane (Spectrum Laboratories) each of the samples was dried and re-suspended in 400 µL of water and treated at 37°C for 5 hours with proteinase K (Sigma). Following this, samples were re-extracted with phenol and dialysed overnight against water as described above. Samples were analysed by 15% SDS-PAGE and stained using Pro-Q emerald glycoprotein stain (Invitrogen).

### Cell walls extractions

Cell walls were extracted from cell pastes (adjusted to approximately 2.5×10^7^ cells in order to normalise our analysis) from each culture time-point. Bacterial cell pastes were re-suspended in 0.3% NaCl and re-fluxed in 50% ethanol at 80°C overnight and spun down at 4000 rpm for 20 minutes. The supernatant was transferred to a glass tube, dialysed overnight, and dried down. To extract the cell walls, the pellets were re-suspended in 5 mL of PBS and sonicated. 5 mL of 4% SDS was then added and this was heated at 85°C for 2 hours. The pellet was washed 3 times with water followed by two washes with 80% acetone and two washes with 100% acetone. This was left to air dry.

### Carbohydrate analysis

Lipoglycans and cell wall material were chemically modified to alditol acetate derivatives as described previously [16] [15]. Gas chromatography (GC) analysis was performed using a Thermoquest Trace GC 2000. Samples were injected in the splitless mode. The column used was a DB225 (Supelco). The oven was programmed to hold at an isothermal temperature of 275°C for a run time of 15 min. All the data were collected and analysed using Xcaliber (v.1.2) software.

### RNA extraction and amplification

Bacterial cell samples were collected from Culture 1 and Culture 2 for RNA extraction throughout the time-course. The sample size depended on the growth phase and ranged between 40–500 mL; larger samples were required later in the culture because of the reduced cell density of the culture (details of samples can be found at http://bugs.sgul.ac.uk/bugsbase, experiment accession no. E-BUGS-142). Each sample was collected directly into 4 volumes of lysis solution (5 M guanidium thiocyanate, 0.5% (w/v) sodium N-lauroyl sarcosine, 25 mM tri-sodium citrate, 0.1 M 2-mercaptoethanol and 0.5% (w/v) Tween 80 in RNAse-free water (Sigma-Aldrich, Poole, UK)) and incubated at room temperature for 1 hour. After centrifugation, the cells were re-suspended in Trizol (Life Technologies Inc., Paisley, UK), and were disrupted for 45 seconds (setting of 6.5) using a ribolyser (Hybaid). RNA was extracted with chloroform and precipitated with isopropanol and 0.3M sodium acetate. The RNA was treated with deoxyribonuclease 1 (DNase I amplification grade, Life Technologies Inc., Paisley, UK) and purified using a Qiagen RNeasy clean up kit (Qiagen, Crawley, UK) following the manufacturer's guidelines. Amplification was performed on 100 ng of total RNA from each time-point, following the method described by the MessageAmp^TM^ II-Bacteria kit (Life Technologies Inc., Paisley, UK) to achieve aRNA concentrations that were approximately 200 fold higher than the starting material.

### Microarray procedures

Three separate labelling reactions were performed on each aRNA sample. Each aliquot of Cy5-labelled cDNA generated from aRNA (test sample) was co-hybridised with Cy3-labelled DNA generated from genomic DNA (control sample). Total RNA (8 µg) was used as a template for reverse transcriptase (Superscript II RNAse H, 200 U µL^−1^; Life Technologies Inc., Paisley, UK) in the presence of random primers and cyanine 5 (Cy5)-labelled dCTP. Genomic DNA was extracted from a cell pellet of *M. tuberculosis* H37Rv harvested from a steady-state culture using the procedure described previously [30]. DNA (1 µg) was used as a template for DNA polymerase (Klenow, 5 U µL^−1^; Life Technologies Inc., Paisley, UK) in the presence of random primers and Cy3-labelled dCTP.

### Array hybridisation

Arrays were performed on 8 time-points for Culture 1 (days 5, 21, 41, 65, 91, 119, 146, and 208) and 6 time-points for Culture 2 (days 2, 3, 16, 23, 85, 222) using whole genome arrays for *M. tuberculosis*; TBv1.1.0 arrays (A-BUGS-1) for Culture 1 and TBv2.1.1 arrays (A-BUGS-23) for Culture 2. The array designs are available in BµG@Sbase (Accession No. A-BUGS-1 http://bugs.sgul.ac.uk/A-BUGS-1 and A-BUGS-23; http://bugs.sgul.ac.uk/A-BUGS-23).

The Cy3 and Cy5 labelled products for each array were combined and purified using a MinElute PCR purification kit (Qiagen). The microarray slides were incubated in pre-hybridisation solution (3.5× SSC, 0.1% (w/v) SDS, bovine serum albumin (BSA 10 mg ml^−1^ Fraction V 96–99%, Sigma-Aldrich) at 65°C for 30 minutes. The slides were rinsed thoroughly in distilled water followed by isopropanol and dried by centrifugation at 1,500 rpm for 5 minutes. The purified Cy3/Cy5 labelled DNA was mixed with hybridisation solution (10.5 µL Cy3/Cy5 labelled DNA, 3.2 µL filtered 20× SSC, and 2.3 µl filtered 2% (v/v) SDS) and heated at 95°C for 2 minutes. The reaction was cooled slightly and centrifuged before being added to the slide and covered with a cover slip. The hybridisation cassette (Telechem International, Sunnyvale, USA) was sealed and submerged in a water-bath at 65°C in the dark for 16–20 hr. After hybridisation, the slides were washed gently in wash solution (1× SSC with 0.05% (w/v) SDS). The slides were rinsed in 0.06× SSC in distilled water and were dried by centrifugation at 1,500 rpm for 5 minutes. Scanning was performed using a dual-laser scanner (Affymetrix 428, MWG-Biotech) at a level (or gain) just below saturation of the most intensely fluorescent spots on each array. The images were quantified using Bluefuse software (https://www.msi.umn.edu/sw/bluefuse).

### Statistical analyses

Transcriptomic analyses were performed on Culture 1 and Culture 2 across the two time-courses. Fully annotated microarray data have been deposited in BµG@Sbase (accession number E-BUGS-142; http://bugs.sgul.ac.uk/E-BUGS-142) and also ArrayExpress (accession number E-BUGS-142). A probabilistic model based on Gaussian process regression and Bayesian model selection was used to analyse and identify consistent genes from which expression profile observations could be merged. The growth curves of Cultures 1 and 2 showed the same dynamics in their growth curves, as determined by total viable count throughout culture (Figure 1). To allow direct comparison of gene expression measurements taken at different time points in each culture, the growth curves were synchronised by applying a linear transformation on the time-scale of one of the cultures. The same transformation was applied to transcriptomic time-points to take the observations onto the same time-scale. The linear transformation was computed by minimising the sum of squared errors between the growth curves of Cultures 1 and 2. The time-point of each RNA sample for Culture 2 was linearly transformed as 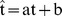
where 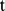 represents the time-point when the culture was sampled and 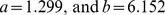.

The observed gene expressions were log-transformed and normalised to zero mean. All replicate measurements were used in the Bayesian model as the probabilistic method takes into account the uncertainty and noise in data, which would be otherwise be lost by averaging. Probabilistic models for gene expression of each gene were created using Gaussian process model [17], which computes a nonlinear regression approximation of the data. A Bayesian model selection procedure was applied to each gene to decide whether the expression profiles for a gene were similar enough in both cultures to be merged into a single time series. Clustering was performed using Bayesian Hierarchical clustering. For technical details see Methods S1 in the supporting information.

The clustering analysis was performed on genes that were identified as being consistent between Cultures 1 and 2. Expression profiles of genes that showed the same expression pattern profile in both cultures were merged together. Replicate expression values were summarised for each time point using a fitted Gaussian process model. Time series clustering of the whole genome data was performed using Bayesian clustering of time series using Gaussian processes with basis function representations, implemented in package SplineCluster [18]. The algorithm automatically determines the optimal number of clusters by maximising likelihoods of different cluster divisions.

Enrichment analysis for clusters was performed using Fisher's exact test for 2×2 contingency matrices as implemented in the R statistical software [63]. Significance of enrichment for an over or under representation of a cluster with respect to a functional class was measured by *p*-values. A cutoff value of *p* = 0.01 was used with the exception of the lipid analysis (GO:0071767 and GO:9999999) for which the cut off was set to 0.05. The *p*-values were not corrected for multiple testing; therefore the enrichment analysis discussed in this work is taken as a heuristic indication of the relevance of certain biological processes. Enrichment was assessed with respect to two different functional annotations. A classification for genes of *M. tuberculosis* has been obtained from the Sanger Institute ftp://ftp.sanger.ac.uk/pub/pathogens/Mycobacterium/tuberculosis/functional_classes/while Gene Ontology http://www.geneontology.org/GO.downloads.ontology.shtml annotations for *M. tuberculosis* were obtained from the MTB GOA project http://www.ark.in-berlin.de/Site/MTB-GOA.html. The Sanger functional annotation provided three levels of increasing detail, through from Level 1 to Level 3. Similar level numbers were also assigned to the Gene Ontology terms.

### Guinea pig aerosol infection

Animals were infected with a low aerosol dose of *M. tuberculosis* H37Rv using a fully contained Henderson apparatus as previously described [64]. Fine particle aerosols of *M. tuberculosis* H37Rv, with a mean diameter of 2 μm (diameter range, 0.5–7 μm) [65], were generated using a Collison nebuliser and delivered directly to the animal snout. The aerosol was generated from a suspension of cells that had been diluted in spent culture medium at each time-point and adjusted to approximately1×10^5^ cfu mL^−1^ in order to obtain an estimated retained, inhaled dose of approximately 10 cfu/lung. The Henderson apparatus allows controlled delivery of aerosols to the animals and the reproducibility of the system and relationship between inhaled cfu and the concentration of organisms in the nebuliser has been described previously [28] [29]. The studies were conducted according to UK Home Office Legislation for animal experimentation and were approved by a local ethical committee at the Health Protection Agency, Porton Down, UK. The project licence number under which the work was completed was PPL 30/2704.

### Bacteriology and histopathology of infected organs

At 16 days and 42 days post-challenge, guinea pigs were killed humanely by intraperitoneal injection of pentabarbitone (Euthatal). Tissues were removed aseptically *post mortem* for bacteriological (cfu counts) and histopathological examination.

Tissues for bacterial counts in organs were homogenised in 10 ml (lungs) or 5 mL (spleens) of sterile distilled water using a rotating blade macerator system (Ystral, UK). Viable counts were performed on the macerate by preparing serial dilutions in sterile water and 100 μl aliquots were plated onto Middlebrook 7H11+ OADC (Oleate, Albumin, Dextrose & Catalase) agar (BioMerieux, UK). Plates were incubated at 37°C for 3 weeks before counting the number of *M. tuberculosis* colonies (cfu).

For histopathological examination, samples of individual lung lobes and spleen were collected, fixed in 10% (v/v) Neutral Buffered Formalin and processed to paraffin wax. Sections cut at 4 μm were stained with haematoxylin and eosin. The nature and severity of the microscopic lesions was evaluated subjectively and scored by a pathologist; evaluations were blinded. Lung lobes were assigned a score as follows: no abnormality  = 0; very small, very few lesions, <10% consolidation  = 1; few or small lesions, 10–20% consolidation  = 2; medium sized lesions, 20–33% consolidation  = 3; moderately sized lesions, 33–50% consolidation  = 4; large lesions, moderately extensive pneumonia, 50–80% consolidation  = 5; extensive pneumonia >80% consolidation  = 6. A mean consolidation score per lobe was calculated for each group. The number of foci of necrosis/caseation and the number of calcified lesions was recorded and a mean score per lobe was calculated for each group. For the spleen, the number of lesions, lesion size and foci of necrosis and calcification were recorded subjectively. For lesion number, >10 = 1, 11–30 = 2, >30 = 4. For lesion size, small lesions  = 1, medium lesions  = 2 and large lesions  = 3. For necrotic and calcified lesions, <5 = 1, 6–10 = 2, >10 = 3.

## Supporting Information

Figure S1 **Depletion of Tween 80 measured in samples of spent supernatant taken from Culture 2 and Culture 3 throughout the time-course.**
(TIF)

Table S1 **Recipe for CMM Mod6 medium.**
(DOCX)

Table S2 **Lipomannan and Lipoarabinomannan biosynthetic genes.**
(DOCX)

Table S3 ***M. tuberculosis*** **genes induced under different** ***in vivo*** **and** ***in vitro*** **conditions.**
(XLSX)

Method S1 **Probabilistic analysis applied to identify consistent genes between the two cultures.**
(DOCX)
