# Supplementary figures and images for: Non-Replicating Mycobacterium tuberculosis Elicits a Reduced Infectivity Profile with Corresponding Modifications to the Cell Wall and Extracellular Matrix
Source: PLoS One. 2014 Feb 6;9(2):e87329. doi: 10.1371/journal.pone.0087329 (PMC3916317; doi:10.1371/journal.pone.0087329)

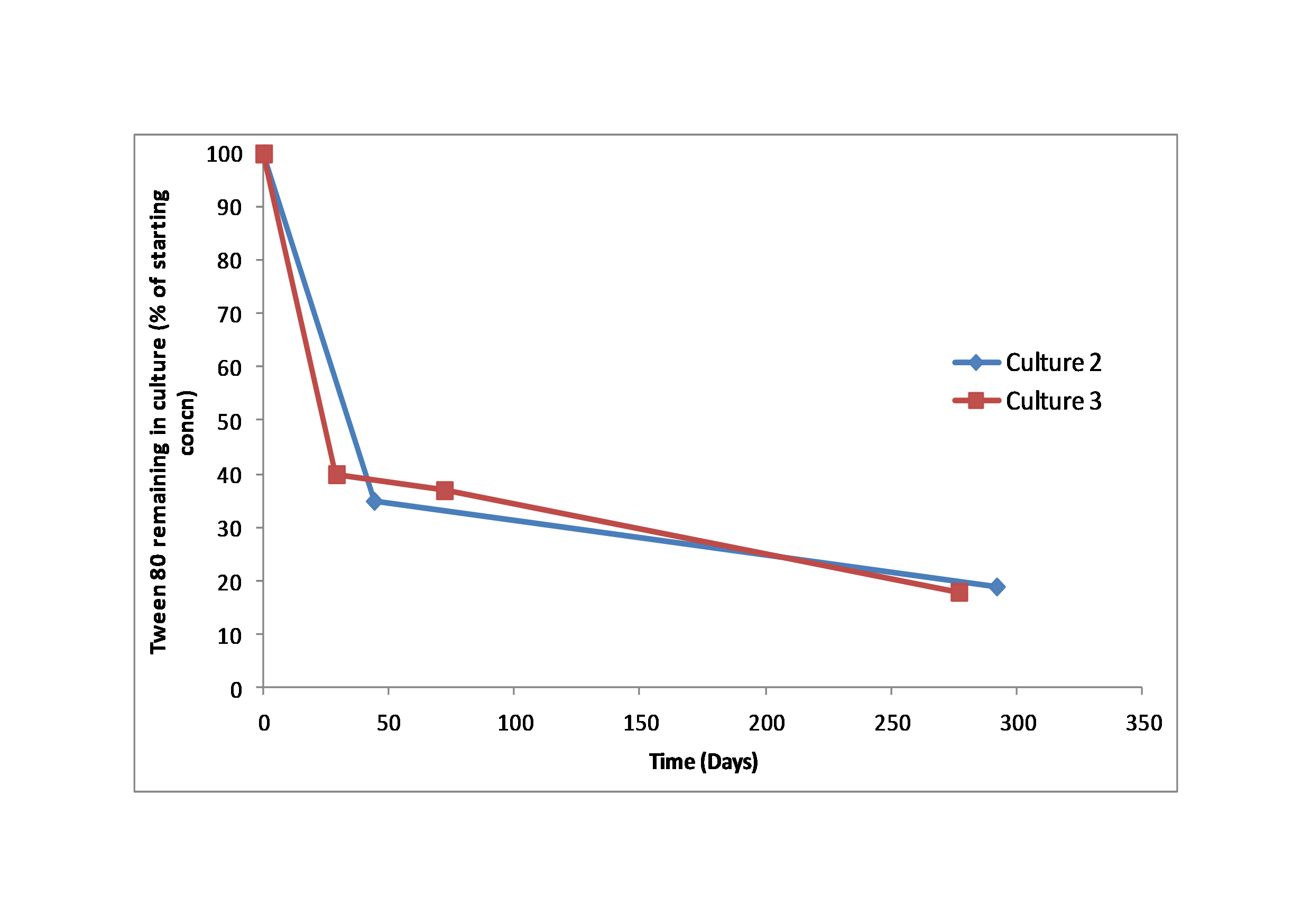

Supplement: Figure S1 — Depletion of Tween 80 measured in samples of spent supernatant taken from Culture 2 and Culture 3 throughout the time-course. (TIF) [file pone.0087329.s001.tif]
