# Supplementary material for: Non-Replicating Mycobacterium tuberculosis Elicits a Reduced Infectivity Profile with Corresponding Modifications to the Cell Wall and Extracellular Matrix
Source: PLoS One. 2014 Feb 6;9(2):e87329. doi: 10.1371/journal.pone.0087329 (PMC3916317; doi:10.1371/journal.pone.0087329)
Supplement: Table S1 — Recipe for CMM Mod6 medium. (DOCX) [file pone.0087329.s002.docx]

| **Medium components** | **Quantity in 1L** |
| --- | --- |
| ***Mod6 Medium*** |  |
| Aces buffer (N-(2-Acetamido)-2-aminoethanesulfonic acid) | 10 g |
| KH2PO4 | 0.22 g |
| Distilled water | 500 mL |
| CYMCO solution 1 | 10 mL |
| CYMCO solution 2 | 10 mL |
| L-asparagine | 2 g |
| Biotin solution (10 µg/mL w/v in water) | 10 mL |
| NaHCO3 | 0.04 g |
| CMYCO Solution 5 | 10 mL |
| Tween 80 | 2 mL |
| Albumin bovine fraction V | 5 g |
| Distilled water | Quantity required to take vol to 1L |
|  |  |
| ***CYCMO solution 1*** |  |
| CaCl_2_.2H_2_O | 0.055 g |
| MgSO_4_.7H_2_O | 21.40 g |
| ZnSO_4_.7H_2_O | 2.88 g |
| Distilled water | 1.0 L |
|  |  |
| ***CYCMO solution 2*** |  |
| CoCl_2_.6H_2_O | 0.048 g |
| CuSO_4_.5H_2_O | 0.0025 g |
| MnCl.4H_2_O | 0.002 g |
| Conc HCL | 0.5 mL |
| Distilled water | 1.0 L |
|  |  |
| ***CYCMO solution 5*** |  |
| FeSO_4_.7H_2_0 | 1.0 g |
| Conc HCL | 0.5 mL |
| Distilled water | 1.0 L |

**Table S1** Recipe for CMM Mod6 medium

|  |
| --- |
| **Method**  Add first two components to the first volume of water. Add the remaining components in the order listed Stir to dissolve Adjust the pH to 6.6 using 20% KOH and check the conductivity. The medium was filter sterilised and store at 4˚C |
