# Supplementary material for: Non-Replicating Mycobacterium tuberculosis Elicits a Reduced Infectivity Profile with Corresponding Modifications to the Cell Wall and Extracellular Matrix
Source: PLoS One. 2014 Feb 6;9(2):e87329. doi: 10.1371/journal.pone.0087329 (PMC3916317; doi:10.1371/journal.pone.0087329)
Supplement: Table S2 — Lipomannan and Lipoarabinomannan biosynthetic genes. (DOCX) [file pone.0087329.s003.docx]

**Table S2** Lipomannan and Lipoarabinomannan biosynthetic genes

| Rv number | Gene name | Function |
| --- | --- | --- |
|  |  | **GDP-Manp biosynthesis** |
| Rv3264c | *manC* | GDP-mannose pyrophosphorylase. Conversion of mannose-1-phosphate to GDP-Manp |
| Rv3255c | *manA* | phosphomannose isomerase converts fructose-6-phosphate to mannose-6-phosphate |
| Rv3257c | *manB* | phophomannomutase converts mannose-6-phosphate to mannose-1-phophate |
|  |  |  |
|  |  | **Synthesis of α-D-mannosyl-1-monophosphoryldecaprenol** |
| Rv2051c | *ppm1* | polyprenyl monophoshomannose synthase |
| Rv3779 |  | transmembrane glycosyltransferase/polyprenyl monophoshomannose synthase trnsferal of galactosamine from polyprenyl-phospho-N-acetylgalactosamine to arabinoglactan in M.tuberculosis |
|  |  |  |
|  |  | **Synthesis of decaprenyl-phospho-arabinose** |
| Rv1449 |  | transketolase links glycolytic and pentose phosphate pathway to give ribose-5-phoshate |
| Rv2465 |  | ribose-5-phosphate isomerase, isomerases D-ribulose-5-phosphate into ribose-5-phosphate |
| Rv1017c | *prsA* | ribose-5-phosphate diphosphokinase converts ribose-5-phosphate into 5-phosphoribosyl-α-1-pyrophosphate (pRpp) |
| Rv3807c |  | phopsholipid phosphatase -in the arabinogalactan biosynthetic cluster |
| Rv3806c | *ubiA* | 5-phospho-α-D-ribose-1-diphosphate:decaprenyl-phosphate 5 phospho-ribosyltransferase |
| Rv3790 |  | 2-OH of ribose of DPR is oxidised of decaprenylphosphoryl-2-keto-β-D-erythro-pentofuranose |
| Rv3791 |  | reduction of decaprenylphosphoryl-2-keto-b-D-erythro-pentofuranose to DPA |
|  |  |  |
|  |  | **Synthesis of phosphatidyl-myo-inositol** |
| Rv0046c |  | glucose-6-phosphate cyclised by inositol-1-phosphate sythase |
| Rv1604 | *impA* | putative inositol monophosphatase |
| Rv2701c | *suhB* | putative inositol monophosphatase |
| Rv2131c | *cysQ* | putative inositol monophosphatase |
| Rv3137 | *impC* | putative inositol monophosphatase |
| Rv2552 |  |  |
|  |  |  |
|  |  | **Conversion of PI into Ac1PIM1** |
| Rv2610c | *pimA* | α-mannopyranosyl-transferase PIM formation from PI and GDP-Manp |
| Rv2611c |  | acyltransferase |
| Rv2609c |  | putative GDP-Manp hydrolase, acylates the 6 position of Manp residue linked to the 2-OH position of myo-inositol |
|  |  |  |
|  |  | **Synthesis of Ac1PIM2** |
| Rv0557 | *mgtA* | α-mannosyl-glucopyranosyluronic acid transferase |
| Rv2188 | *pimB* | α-D-mannose-α(1→6)-phosphatidyl-myo-inositol-mannopyranosyltransferase |
| Rv2190c |  | PIM or PPM transport |
|  |  |  |
|  |  | **Synthesis of higher order PIMs** |
| Rv1159 | *pimE* | Elongation, branching of lipomannan and liparabinomannan, α(1→2)-mannopyronosyltransferase |
| Rv0051 |  | Glycosyltransferase-PimE? |
| Rv0541c |  | Glycosyltransferase-PimE? |
| Rv1166 | *lpqW* | Channels Ac1/Ac2PIM4 for lipomannan synthesis |
| Rv1565c |  | Acyl transferase, acylation of higher order PIM, LAM, LM |
|  |  |  |
|  |  | **Synthesis of the mannan core of LM and LAM biosynthesis** |
| Rv2174 | *mptA* | α-(1→6)-mannopyranosyltransferase |
| Rv1459c | *mptB* | α-(1→6)-mannopyranosyltransferase |
| Rv1458c |  | ABC transporter? |
| Rv1457c |  | ABC transporter? |
| Rv1456c |  | ABC transporter? |
| Rv2173 | *idsA2* | putative geranylgeranyl pyrophosphate synthetase |
| Rv2181 | *mptC* | synthesis of α(1→2)-Manp side chains of lipomannan |
|  |  |  |
|  |  | **Arabinan domain assembly of LAM** |
| Rv3793 | *embC* | α(1→5)-arabinofuranosyltransferase |
| Rv2673 | *aftC* | α(1→3) arabinofuranosyltransferase |
| Rv0236c | *aftD* | α(1→3) or α(1→5)-arabinofuranosyltransferase |
| Rv3805c | *aftB* | α(1→2)-arabinofuranosyltransferase |
| Rv1635c | *capA* | α(1→5)-Mannopyranosyltransferase |
