# Supplementary material for: Non-Replicating Mycobacterium tuberculosis Elicits a Reduced Infectivity Profile with Corresponding Modifications to the Cell Wall and Extracellular Matrix
Source: PLoS One. 2014 Feb 6;9(2):e87329. doi: 10.1371/journal.pone.0087329 (PMC3916317; doi:10.1371/journal.pone.0087329)
Supplement: Method S1 — Probabilistic analysis applied to identify consistent genes between the two cultures. (DOCX) [file pone.0087329.s005.docx]

**Method S1** Probabilistic analysis applied to identify consistent genes between the two cultures

***Bayesian analysis***

Probabilistic model for gene expression of each gene was created using Gaussian process model **[ref1]**, which computes a nonlinear regression approximation of the data. Gaussian process is defined as a probability distribution over functions

where $\mathbf{t}=(t_{1},\ldots,t_{T})^{T}$are the transcriptomics time points, $m(\mathbf{t})$is a mean function and $k(\mathbf{t},\mathbf{t}')$is a covariance function of the Gaussian process. The covariance function governs the expected smoothness of the gene expression model in time. Covariance function used in our application is the squared exponential with diagonal noise term

$K_{ij}=k(t_{i},t_{j})=\sigma_{f}^{2}\exp\left\{-\frac{1}{2l^{2}}(t_{i}-t_{j})^{2}\right\}+\sigma^{2}\delta_{ij}$,

where $\sigma^{2}$is the noise variance, $\delta_{ij}=1\text{ if }i=j\text{ and is equal to }0\text{ otherwise}$, $\sigma_{f}\text{ and }l$ together with $\sigma$are the so-called hyperparameters of the covariance function. By using the squared exponential covariance function with appropriate priors on the hyperparameters, we assume smooth expression changes for each gene over time. For this model, the hyperparameters were given log-normal priors: $\log(l)\sim N(4.6,1.1)$, $\log(\sigma_{f})\sim N(0,0.1)$ and $\log(\sigma)\sim N(0.9,2)$.

For a finite set of observed gene expressions $\mathbf{x}$at time points$\mathbf{t}$, the corresponding sample from a Gaussian process has a regular multivariate normal distribution

,

where $K(\mathbf{t},\mathbf{t}')$is the covariance matrix evaluated at T observation time points $\mathbf{t}$,$K_{ij}=k(t_{i},t_{j}),i,j=1,\ldots,T$.

Given the observed expression values for each gene and prior distributions over hyper-parameters, posterior distribution over functions is then computed.

By working in a probabilistic framework, it is possible to compute marginal likelihood of observed data given a model by integrating out the hyperparameters of the model. We want to distinguish two probabilistic models of the gene expression data, the first one represents the consistent genes and the second one represents the genes that cannot be merged. Observations of genes that behave consistently in culture 1 and culture 2 are assumed to come from a single underlying generative model in both cultures. If observations behave differently in culture 1 and culture 2, they are modelled as coming from two distinct underlying models.

Model 1: gene behaves consistently in culture 1 and 2.

Model 2: gene shows different behaviour in culture 1 than in culture 2.

Likelihoods of the two possible models are then compared using the Bayes factor

$B=\frac{p(M_{1}|\mathbf{t}^{A},\mathbf{t}^{C},\mathbf{x}^{A},\mathbf{x}^{C})}{p(M_{2}|\mathbf{t}^{A},\mathbf{t}^{C},\mathbf{x}^{A},\mathbf{x}^{C})}=\frac{p(\mathbf{x}^{A},\mathbf{x}^{C}|\mathbf{t}^{A},\mathbf{t}^{C},M_{1})p(M_{1})}{p(\mathbf{x}^{A}|\mathbf{t}^{A},M_{2})p(\mathbf{x}^{C}|\mathbf{t}^{C},M_{2})p(M_{2})}$,

where $p(M_{i}|\mathbf{t},\mathbf{x})$ is the likelihood of model $M_{i}$ given observed gene expressions at given time points. Prior probabilities over models were assumed to be equal, $p\left( M_{1} \right)= p\left( M_{2} \right)= 0.5$. Marginal likelihoods $p\left( \mathbf{x} \right|\mathbf{t},M_{i})$ are not analytically tractable and were approximated using hybrid Monte Carlo sampling **[ref2]**.

Values of Bayes factor larger than 1 support model$M_{1}$, i.e. a given gene shows consistent behaviour in cultures 1 and 2. On the other hand, values smaller than 1 indicate that the two separate models are more probable.

The Gaussian process regression and Bayesian model selection was implemented in Matlab.

***Clustering analysis***

The clustering analysis was performed separately on genes that were identified as being consistent between cultures 1 and 2, and on genes that showed different behaviour between the cultures. Expression profiles of genes that showed the same behaviour in both cultures were merged together. Replicate expression values were summarized for each time point using a fitted Gaussian process model.

Time series clustering of the whole genome data was performed using Bayesian clustering of time series using Gaussian processes with basis function representations implemented in package SplineCluster by N. A. Heard.

Clustering of the lipid genes was performed using Bayesian Hierarchical clustering, available in R/Bioconductor package BHC. This algorithm yields more plausible solutions for smaller sets of genes than SplineCluster. The BHC clustering method is more computationally demanding due to approximate inference using MCMC and therefore it was used only to cluster a limited number of genes, i.e. the genes of primary interest.

Both algorithms automatically determine the optimal number of clusters by maximizing likelihoods of different cluster divisions.

[ref1] C. E. Rasmussen and C. K. I. Williams. *Gaussian Processes for Machine Learning*. The MIT Press, Cambridge, MA, 2006.

[ref2] S Duane, AD Kennedy, and BJ Pendleton. *Hybrid Monte Carlo*. Physics letters B, 1987.
